# Sustainable Synthesis of Faujasite-Type Zeolites Synthesized from Rice Husk for Hg^2+^ Removal from Aqueous Solutions: Adsorption Performance, Mechanistic Insights, and Environmental Safety Assessment

**DOI:** 10.3390/molecules31173101

**Published:** 2026-09-04

**Authors:** Naren Bocanegra, Marcela Paredes-Laverde, Nancy Acelas, Ximena Carolina Pulido, Luis Rodríguez, César Jaramillo-Páez

**Affiliations:** 1 Grupo de Investigación en Química Aplicada a Procesos Ecológicos (QUAPE-UT), Facultad de Ciencias, Universidad del Tolima, Ibagué 730006, Colombia; nbocanegram@ut.edu.co (N.B.); xpulido@ut.edu.co (X.C.P.); lfrodriguezh@ut.edu.co (L.R.); 2 Grupo de Investigación Navarra Medicina, Facultad de Ciencias de la Salud, Fundación Universitaria Navarra-Uninavarra, Neiva 410010, Colombia; m.paredes@uninavarra.edu.co; 3 Grupo de Investigación Materiales con Impacto (MAT&MPAC), Facultad de Ingenierías, Universidad de Medellín, Medellín 050026, Colombia

**Keywords:** mercury removal, faujasite zeolite, rice husk ash, adsorption, ecotoxicity, water treatment

## Abstract

Rice husk, an abundant agro-industrial by-product rich in SiO_2_, represents a promising precursor for the sustainable synthesis of zeolites. In this study, rice husk ash was used to synthesize faujasite-type X and faujasite-type Y, and their performance for Hg^2+^ removal from aqueous solutions was comparatively evaluated. X-ray diffraction confirmed the successful formation of the faujasite structures, while physicochemical characterization revealed differences in pore structure and surface chemistry. FAU-type X exhibited higher Hg^2+^ removal than FAU-type Y, consistent with the combined influence of its lower Si/Al ratio, higher framework charge density and ion-exchange capacity, as well as its larger pore volume and average pore diameter. Based on its higher Hg^2+^ removal, FAU-type X was selected for a comprehensive evaluation of its adsorption performance and applicability under environmentally relevant conditions. The pseudo-second-order model best described adsorption kinetics for both zeolites, whereas thermodynamic analyses indicated that the adsorption process was spontaneous and endothermic. Optimal adsorption conditions for FAU-type X were achieved at pH 6.8, using an adsorbent dosage of 0.75 g L^−1^, a contact time of 24 h, and an initial Hg^2+^ concentration of 1 mg L^−1^. Equilibrium data were best fitted by the Sips isotherm model, indicating adsorption on a heterogeneous surface with a maximum adsorption capacity of 83.14 mg g^−1^. FAU-type X retained appreciable adsorption performance after four regeneration cycles, although Hg^2+^ removal efficiency decreased in Caquetá River water because of competition from coexisting metal ions. To assess the environmental implications of the treated water beyond Hg^2+^ removal efficiency, ecotoxicological assays demonstrated the sensitivity of *Daphnia magna* to residual Hg^2+^ concentrations, whereas reductions in *Escherichia* coliforms were mainly attributed to the adsorption process. In addition, *Lactuca sativa* seedlings exhibited approximately 50% inhibition of elongation after treatment. Overall, these findings demonstrate the potential of rice husk-derived faujasite-type X as a sustainable adsorbent for Hg^2+^ removal, while highlighting the need for complementary treatment strategies to ensure the environmentally safe discharge of water and its agricultural reuse.

## 1. Introduction

Mercury (Hg) contamination remains a major environmental concern due to its persistence, toxicity, bioaccumulation, and biomagnification in aquatic ecosystems [1,2]. Anthropogenic activities such as mining, fossil fuel combustion, industrial discharges, and the improper disposal of mercury-containing products have significantly increased mercury emissions into the environment [3]. In aquatic systems, inorganic mercury can be adsorbed onto suspended particles and sediments, where anaerobic microorganisms may convert it into methylmercury, the most toxic and bioavailable form of mercury [4]. Methylmercury readily accumulates in aquatic organisms and biomagnifies throughout the food chain, making fish consumption the primary route of human exposure [5]. Consequently, mercury contamination poses serious risks to human health and aquatic ecosystems, including neurotoxicity, developmental disorders, reproductive impairment, and ecological disruption [6,7].

Various technologies have been investigated for mercury removal from contaminated water, including precipitation, coagulation, ultrafiltration, solvent extraction, photocatalysis, ion exchange, and adsorption [8]. Among these approaches, adsorption has attracted particular attention for its operational simplicity, low cost, high removal efficiency, and the wide availability of environmentally friendly adsorbents [9]. In this context, zeolites have emerged as promising adsorbents due to their well-defined microporous aluminosilicate framework, composed of interconnected TO_4_ tetrahedra (T = Si or Al) linked through oxygen atoms [10]. Zeolites can be obtained from natural mineral deposits or synthesized under controlled hydrothermal conditions, enabling the production of materials with tailored physicochemical properties for specific environmental applications [11]. Their high specific surface area, tunable pore structure, excellent chemical and thermal stability, and abundant ion-exchange sites confer a strong affinity for heavy-metal ions, making them attractive materials for sustainable water treatment technologies [12,13].

Among zeolitic materials, FAU-type zeolites have attracted particular interest because of their three-dimensional porous network, large pore volume, and high ion-exchange capacity [14]. Faujasites with Si/Al ratios between 1 and 1.5 are generally classified as type X zeolites, whereas those with Si/Al ratios above 1.5 are classified as type Y zeolites [15]. Although these materials can be synthesized with tailored physicochemical properties, using conventional chemical precursors may increase production costs and reduce the sustainability of the synthesis process. Consequently, considerable effort has focused on replacing conventional silica sources with low-cost and renewable alternatives [16]. In this regard, rice husk (Oryza sativa), an abundant agro-industrial by-product representing approximately 20–25% of the total grain weight, has emerged as a promising precursor for zeolite synthesis [17]. Rice husk contains significant amounts of cellulose, lignin, and silica (15–17%) [18], and after thermal treatment, the resulting rice husk ash (RHA) may contain up to 85–95% amorphous silica [19]. Consequently, RHA has been successfully employed as a sustainable and economically attractive source of silica for the synthesis of FAU-type zeolites. Previous studies have demonstrated the applicability of rice husk-derived zeolites for the removal of toxic heavy metals, including Zn, Hg, Cd, Cr, and Pb, from contaminated wastewater [20,21].

Despite increasing interest in FAU-type materials for heavy-metal remediation, direct comparisons of the adsorption performance of rice husk-derived faujasites X and Y remain scarce. In particular, it remains unclear whether differences in their framework compositions, textural properties, and surface chemistry result in distinct Hg^2+^ adsorption behaviors and mechanisms. Furthermore, most studies have evaluated mercury removal under simplified conditions using synthetic solutions, whereas the performance of these materials in natural water matrices remains poorly understood. In addition, the environmental safety of treated water, particularly with respect to ecotoxicological, microbiological, and phytotoxicological impacts, has received limited attention. Therefore, an important knowledge gap remains regarding the relationship among the physicochemical properties of rice husk-derived faujasites, their adsorption performance, and the environmental implications of their practical application.

Therefore, this study aimed to synthesize faujasite-type X and faujasite-type Y from rice husk ash and to comparatively evaluate their performance in removing Hg^2+^ from aqueous solutions. Both materials were characterized by their chemical composition, crystalline structure, morphology, surface chemistry, and textural properties. Based on the kinetic and thermodynamic results, the zeolite with the most favorable Hg^2+^ adsorption performance was selected for further investigation. Subsequently, the effects of initial pH, adsorbent dosage, and initial Hg^2+^ concentration were analyzed. Complementarily, adsorption isotherms and material reusability were assessed, and a plausible adsorption mechanism was proposed. In addition, the environmental safety of the treated water was assessed through ecotoxicological analyses using *Daphnia magna*, *Lactuca sativa*, *E. coli*, and total coliforms. This integrated approach provides new insights into the relationship among the physicochemical properties of rice husk-derived faujasites, their Hg^2+^ adsorption performance, and the environmental implications of their use in sustainable water treatment applications.

## 2. Results and Discussion

### 2.1. Ability of Zeolites to Remove Hg^2+^ in Water and Its Relationship with Physicochemical Properties

**Figure 1:**
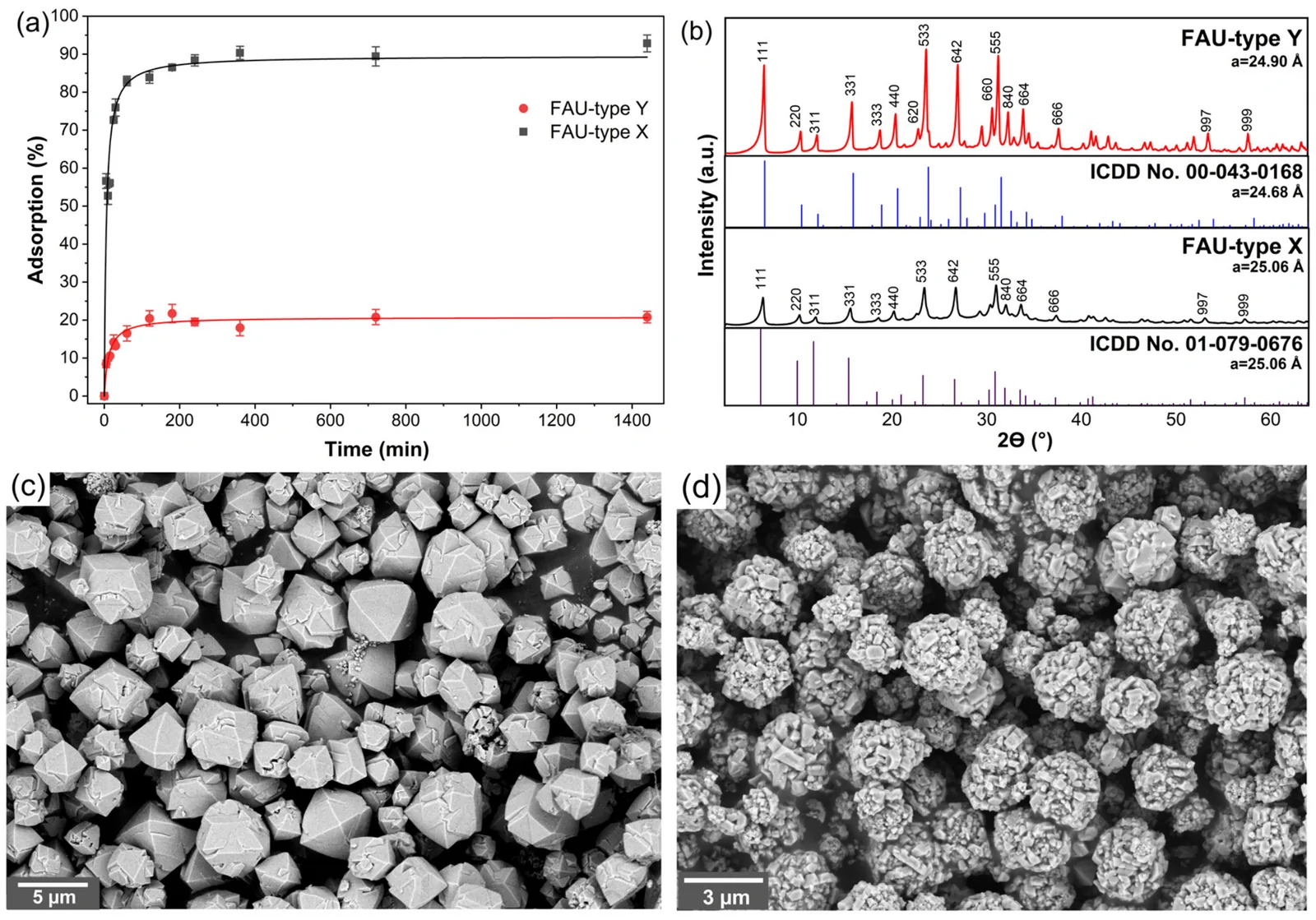
Evaluation of Zeolite materials: (**a**) Removal of Hg^2+^ present in distilled water using FAU-type Y and FAU-type X as adsorbents. Conditions: pH 6.8, initial concentration of Hg^2+^ 1.0 mg L^−1^, stirring rate 270 rpm, adsorbent dose 1 g L^−1^, temperature 25 °C. (**b**) Characterization XRD. SEM micrographs: (**c**) FAU-type Y and (**d**) FAU-type X.

Faujasite-type zeolites have been extensively investigated as adsorbents for removing contaminants from aqueous systems due to their high ion-exchange capacity and tunable porous structure. In this study, the adsorption performance of rice husk-derived faujasite X and faujasite Y toward Hg^2+^ removal was comparatively evaluated. As shown in Figure 1a, FAU-type X achieved a Hg^2+^ removal efficiency of 89% at equilibrium (720 min), whereas FAU-type Y removed only 21% under the same conditions. Furthermore, no evidence of Hg^2+^ desorption was observed up to 1440 min, indicating the stability of the adsorption process. Notably, even as a preliminary result, the removal efficiency achieved by FAU-type X is comparatively high, to report for the same faujasite unmodified and doped with Ag [22]. To elucidate the superior performance of FAU-type X, both zeolites were comprehensively characterized with respect to their surface chemistry, crystalline structure, textural properties, and morphology. The FTIR spectra of FAU-type Y and FAU-type X revealed similar functional groups (Supplementary Material (S) Figure S1), and the corresponding assignments are summarized in Table 1. A broad absorption band centered at 3347 cm^−1^ was attributed to the O–H stretching vibrations of silanol groups and adsorbed water molecules. The presence of molecular water was further corroborated by the H–O–H bending vibration at 1646 cm^−1^ [23], whereas the band at 962 cm^−1^ was assigned to Si–O stretching vibrations associated with silanol groups [24]. Additional bands located at 744 and 663 cm^−1^ were attributed to the symmetric stretching vibrations of Si–O–Al and Si–O–Si bonds, respectively, confirming the formation of an aluminosilicate framework composed of interconnected SiO_4_ and AlO_4_ tetrahedra [25]. Moreover, the characteristic band at 558 cm^−1^, assigned to the double six-membered rings (D6R) units, corroborated the successful synthesis of the faujasite structure [26]. These observations are consistent with the Si and Al contents determined by EDS analysis (Table S1).

Thus, for FAU-type Y and FAU-type X, in addition to the identified functionalities, the surface charge properties of the zeolites should also be considered to explain the observed Hg^2+^ removal behavior. Both materials exhibited pH_pzc_ values close to 9 (Figure S2 and Table 1). These findings are consistent with reports in the literature, where pH_pzc_ values between 9.3 and 9.9 have been reported for synthesized FAU-type X samples [27]. A value of 8.0 has been reported for FAU-type Y with a Si/Al ratio of 2.3 [28], compared with a Si/Al ratio of 1.61 for FAU-type Y in the present study. Although the Si/Al ratio influences the framework charge of FAU-type zeolites, the experimentally determined pH_pzc_ cannot be attributed solely to framework composition. Surface hydroxyl groups, the nature and distribution of charge-compensating cations, and synthesis-dependent surface properties may also influence the measured pH_pzc_. Therefore, the small difference observed between FAU-type X (9.27) and FAU-type Y (9.08) likely reflects the combined contribution of these factors rather than a direct dependence on the Si/Al ratio alone. Under the experimental conditions (pH 6.8), surface silanol groups (pKa ≈ 4.5) [28] are expected to be predominantly deprotonated, generating negatively charged Si–O^−^ sites capable of interacting with Hg^2+^ species through electrostatic attraction and surface complexation mechanisms. Additionally, isomorphic substitution of Si^4+^ by Al^3+^ within the framework generates permanent negative charges that are compensated by exchangeable cations, thereby promoting Hg^2+^ uptake via ion-exchange processes [29]. Although both zeolites exhibited similar functional groups, their relative intensities were consistently higher for FAU-type X, particularly for the band at 962 cm^−1^ assigned to silanol groups (Figure S1 and Table 1).

In addition, EDS elemental mapping (Table S1) confirmed the local presence and distribution of Si, Al, and Na in both zeolites. The semi-quantitative EDS-derived Si/Al molar ratios were 1.13 for FAU-type X and 1.61 for FAU-type Y, consistent with the expected compositional difference between the two faujasite materials [30]. XRF analysis (Table S4) provided complementary information on the bulk chemical composition, confirming Si and Al as the predominant framework elements and yielding Si/Al ratios of 2.33 for FAU-type X and 3.10 for FAU-type Y. Si/Al ratios above 1.5 have been reported for faujasite synthesized using rice husk-derived silica when the composition was determined by X-ray fluorescence (XRF) [31]. This apparent discrepancy with the conventional compositional range of FAU-type X can be attributed to the contribution of a residual amorphous silica fraction originating from the rice husk [32]. Although this fraction is not incorporated into the crystalline framework, it is detected by XRF, thereby increasing the overall Si content measured in the sample. Although the absolute Si/Al values obtained by EDS and XRF differ, both techniques consistently indicate a lower Si/Al ratio for FAU-type X than for FAU-type Y. These differences arise from the distinct analytical principles and sampling volumes of the two techniques: EDS provides localized elemental information from specific regions of the sample, whereas XRF provides a more representative bulk composition. Therefore, the EDS and XRF results are considered complementary rather than directly interchangeable. The lower Si/Al ratio of FAU-type X is consistent with a higher framework negative charge density and, consequently, a greater number of charge-compensating cations potentially available for ion exchange with Hg^2+^. Together with the higher relative abundance of silanol groups observed for FAU-type X, these compositional and surface characteristics likely contribute to its enhanced Hg^2+^ adsorption through the combined effects of electrostatic interactions, surface complexation, and ion-exchange processes.

To complement the surface characterization results and determine whether structural differences contributed to the observed adsorption behavior, X-ray diffraction (XRD) analysis was performed (Figure 1b). The diffraction patterns of FAU-type X and FAU-type Y showed good agreement with the reference cards ICDD No. 01-079-0676 and ICDD No. 00-043-0168, respectively, with no additional reflections attributable to secondary crystalline phases detected. To further support the phase identification presented in Figure 1b, the unit-cell parameters (a0) of the synthesized faujasites have been included. FAU-type X exhibited a unit-cell parameter of 25.06 Å, which exactly matches the value reported in the ICDD PDF No. 01-079-0676. In contrast, FAU-type Y showed a value of 24.90 Å, which is close to the 24.68 Å reported in ICDD PDF No. 00-043-0168. Although some reflections, particularly the (111) reflection at approximately 6.2–6.4° (2θ), generally exhibit higher relative intensities in FAU-type zeolites [33], variations in peak intensity have been reported depending on synthesis conditions, including acid concentration during precursor pretreatment and crystallization temperature [34]. Both materials exhibited diffraction peaks over a 2θ range of 6–60°, with corresponding interplanar spacings (d) ranging from 1.53 to 15 Å (Table S2), supporting the successful formation of highly crystalline FAU-type frameworks. While Figure 1b shows only the main Miller indices common to both faujasites, Table S2 lists the complete reflection set. These additional, lower-intensity reflections, along with slight shifts in diffraction angles that are difficult to distinguish by visual inspection alone, provide the basis for distinguishing the two phases. Therefore, while XRD supports the formation of the FAU framework in both materials, their markedly different Hg^2+^ adsorption behavior should be interpreted considering the combined influence of framework composition, particularly the Si/Al ratio and associated ion-exchange capacity, together with their textural and morphological properties.

To further investigate the origin of the different adsorption performances, N_2_ physisorption analysis was conducted. The adsorption–desorption isotherm of FAU-type Y (Figure S3) was classified as type I with an H4 hysteresis loop, indicating a zeolitic structure characterized by pores predominantly distributed near the upper limit of the micropore range (≈2 nm), together with small mesopores exhibiting narrow slit-shaped geometries [35]. FAU-type X exhibited a type IV adsorption–desorption isotherm with an H3 hysteresis loop (Figure S3), indicating the presence of mesoporosity within the material [36]. This observation is consistent with the textural parameters summarized in Table 1, which show that FAU-type X has a larger average pore diameter and higher pore volume than FAU-type Y. In contrast, FAU-type Y exhibited a higher specific surface area (S_BET_ of 679 m^2^ g^−1^), consistent with values reported for conventional faujasite Y materials (660 m^2^ g^−1^ [37] and 639 m^2^ g^−1^ [38]). In contrast, FAU-type X showed a lower S_BET_ (488 m^2^ g^−1^), comparable to previously reported values for faujasite X zeolites (453 m^2^·g^−1^ [39] and 486 m^2^ g^−1^ [40]). However, the BET surface area is not governed by the Si/Al ratio alone. The higher framework charge associated with the lower Si/Al ratio of FAU-type X requires a greater concentration of charge-compensating extra-framework cations, whose presence and distribution within the pore system may affect N_2_ accessibility during physisorption measurements. In addition, the measured surface area is influenced by pore structure, pore volume, crystallinity, and synthesis conditions. Despite its higher S_BET_, FAU-type Y exhibited substantially lower Hg^2+^ removal than FAU-type X, demonstrating that adsorption performance is not determined by specific surface area alone. The superior adsorption behavior of FAU-type X can instead be associated with the combined influence of framework composition and pore accessibility. Its lower Si/Al ratio results in a higher negative framework charge and, consequently, a greater density of charge-compensating cations potentially available for ion-exchange interactions with Hg^2+^. In addition, the larger pore volume and average pore diameter of FAU-type X may facilitate Hg^2+^ transport and accessibility to adsorption sites within the zeolite structure. Therefore, the contrasting adsorption behavior of FAU-type X and FAU-type Y highlights the importance of the chemical nature and accessibility of active sites, rather than S_BET_ alone, in controlling Hg^2+^ adsorption.

The morphology of the synthesized zeolites was further investigated by SEM. FAU-type Y exhibited well-defined octahedral particles (Figure 1c), whereas FAU-type X displayed spheroidal and irregular aggregates formed by the sheet agglomeration of smaller particles (Figure 1d), morphologies consistent with those reported by other authors for FAU-type Y and FAU-type X zeolites, respectively [41,42]. Although SEM observations alone do not establish adsorption performance, the more heterogeneous and aggregated morphologies of FAU-type X may improve accessibility to adsorption sites [43] and facilitate mass-transfer processes within the material. These observations suggest that morphological characteristics, together with surface chemistry and textural properties, may influence Hg^2+^ adsorption behavior.

Overall, these results indicate that the superior Hg^2+^ adsorption performance of FAU-type X compared with FAU-type Y arises from the synergistic contribution of several physicochemical properties, including a higher abundance of active surface sites, particularly Si–OH and Si–O–Al groups, together with a larger average pore diameter, higher pore volume, and more heterogeneous morphology. These findings demonstrate that Hg^2+^ adsorption by FAU-type zeolites is governed by the accessibility and chemical nature of adsorption sites rather than by specific surface area alone. Nevertheless, a comprehensive understanding of the adsorption process also requires kinetic and thermodynamic analyses, which are discussed in the following section.

### 2.2. Kinetics and Thermodynamic Analysis

**Figure 2:**
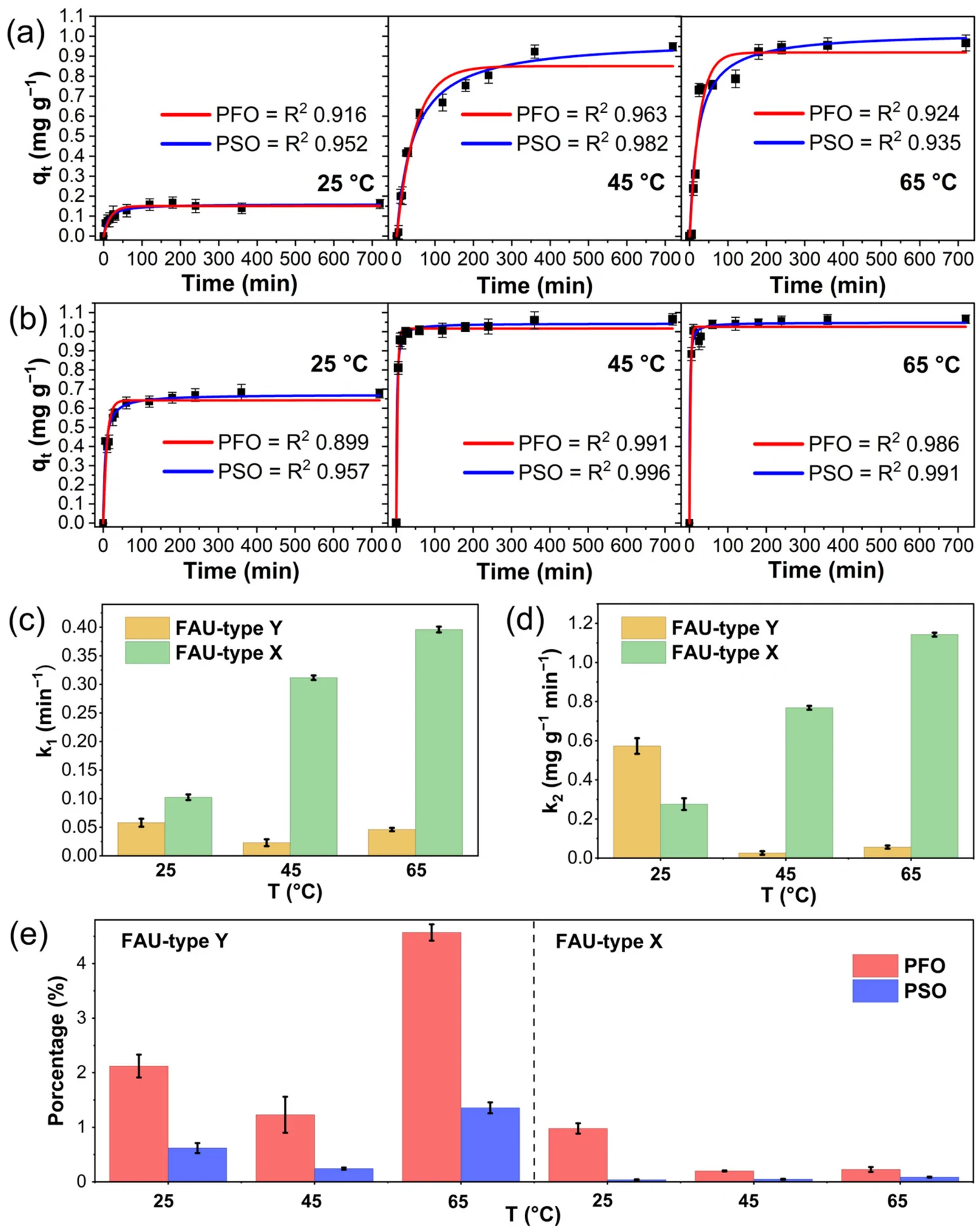
Effect of temperature on adsorption capacity. (**a**) qt for FAU-type Y; (**b**) qt for FAU-type X. (**c**) k_1_ and (**d**) k_2_. (**e**) APE. Conditions: pH 6.8, initial concentration of Hg^2+^ 1.0 mg L^−1^, stirring rate 270 rpm, adsorbent dose 1 g L^−1^.

The adsorption kinetics of Hg^2+^ onto FAU-type Y and FAU-type X were evaluated using the pseudo-first-order (PFO) and the pseudo-second-order (PSO) models at 25, 45, and 65 °C (Figure 2a,b). As shown in Figure 2, the adsorption capacity (q_t_) increased from 25 to 45 °C for both zeolites and remained nearly constant at 65 °C, with consistently higher adsorption capacities observed for FAU-type X than for FAU-type Y. In contrast, the kinetic rate constants exhibited different temperature-dependent behaviors. The PFO rate constant (k_1_) decreased with increasing temperature for FAU-type Y but increased for FAU-type X (Figure 2c). A similar trend was observed for the PSO rate constant (k_2_) (Figure 2d). These results indicate that temperature exerts a greater influence on the adsorption rate than on the adsorption capacity. Therefore, the fitting performance of both kinetic models was evaluated to determine the most appropriate description of the Hg^2+^ adsorption process.

Although both kinetic models yielded satisfactory fits (R^2^ ≥ 0.90) for Hg^2+^ adsorption onto FAU-type Y and FAU-type X, the PSO model consistently provided higher correlation coefficients and lower average percentage errors (APEs) than the PFO model (Figure 2e). The APE values remained below 5% for both zeolites, further supporting the PSO model’s superior predictive capability. Taken together, these findings indicate that the PSO model over the investigated temperature range better describes Hg^2+^ adsorption onto FAU-type Y and FAU-type X. The good agreement with the PSO model suggests that adsorption kinetics are mainly governed by the availability of active sites on the adsorbent surface rather than by external mass-transfer limitations [44]. For FAU-type X, the increase in the kinetic constants with temperature suggests that thermal energy facilitates the migration of Hg^2+^ ions toward the active sites, resulting in faster adsorption kinetics. In contrast, the decrease in adsorption rate observed for FAU-type Y may indicate that diffusion limitations within its predominantly microporous structure become more significant at elevated temperature. These contrasting kinetic behaviors are consistent with the differences in surface chemistry and textural properties discussed in Section 2.1.

Although kinetic analyses provide information regarding the adsorption rate and possible controlling mechanisms, they do not establish the thermodynamic feasibility of the process. Therefore, the thermodynamic parameters ΔH°, ΔG°, and ΔS° were determined for FAU-type Y and FAU-type X over the temperature range of 25–65 °C using a linear relationship between ln Kc and 1/T (Figure S4), and the obtained values are reported in Table 2. Both zeolites exhibited positive ΔH° values, indicating that Hg^2+^ adsorption is an endothermic process. Moreover, the negative ΔG° values confirm the spontaneous nature of adsorption, with spontaneity becoming more favorable as temperature increases [45]. The positive ΔS° values suggest an increase in disorder at the solid–liquid interface during adsorption. Notably, FAU-type X exhibited lower ΔH° and ΔS° values, along with more negative ΔG° values, than FAU-type Y, indicating a thermodynamically more favorable adsorption process that requires less energy and involves a lower degree of interfacial reorganization.

Furthermore, the relatively low ΔH° values obtained for both zeolites, particularly for FAU-type X (5.32 kJ mol^−1^), suggest that Hg^2+^ adsorption is predominantly governed by weak interactions, such as electrostatic attraction, ion-exchange processes, and dipole interactions, rather than by strong chemical bonding. These findings are consistent with the adsorption mechanisms proposed from the physicochemical characterization analyses. Considering its superior adsorption capacity, enhanced kinetic performance, and more favorable thermodynamic parameters, FAU-type X was selected as the most promising adsorbent for the subsequent evaluation of operational conditions.

### 2.3. Effects of FAU-Type X Dose, Initial Concentration of Pollutant, and pH Solution

**Figure 3:**
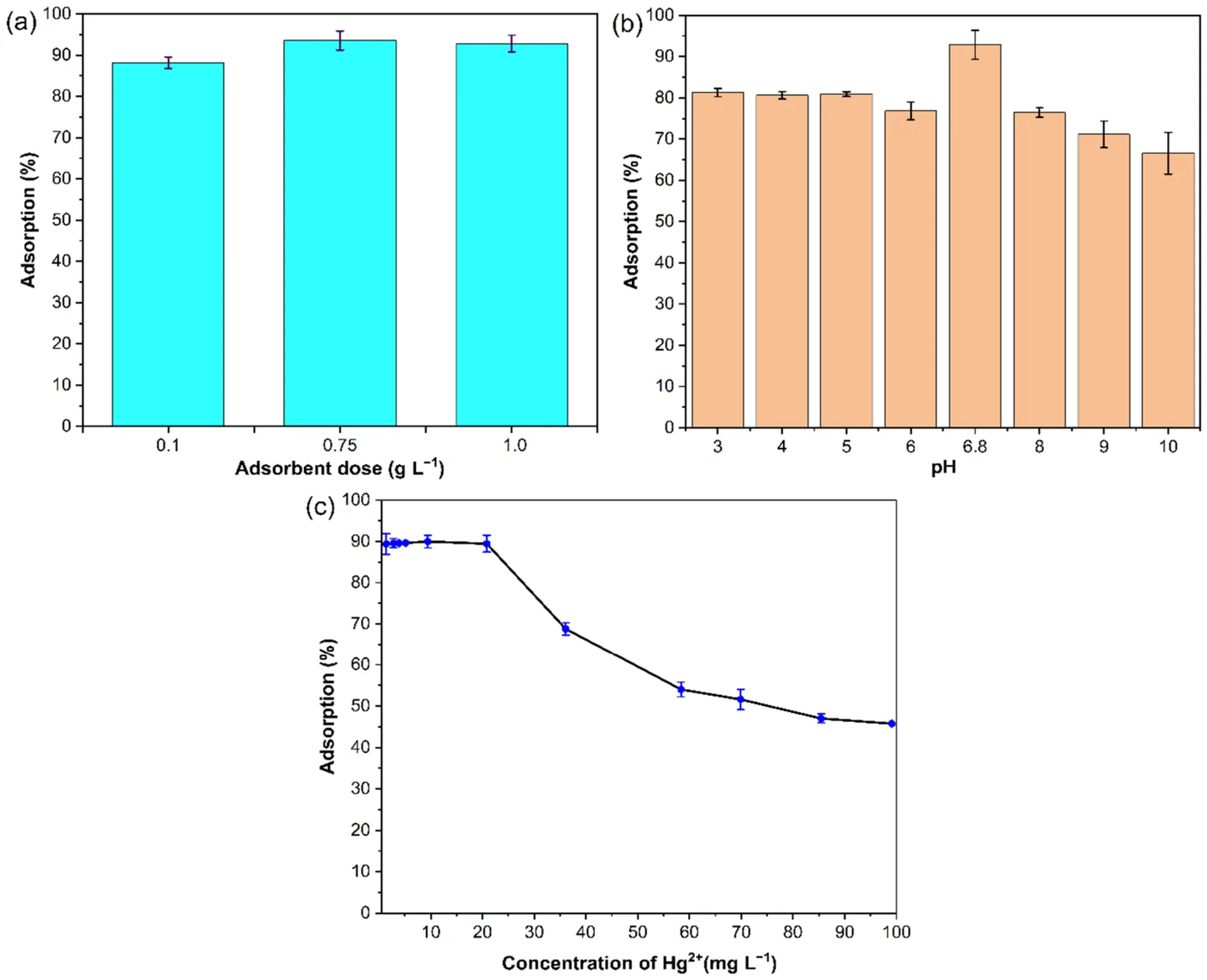
Influence of key variables on the adsorption of Hg^2+^. (**a**) Effect of adsorbent dosage on percent mercury removal. Conditions: pH 6.8, initial concentration of Hg^2+^ 1.0 mg L^−1^, stirring rate 270 rpm, time 24 h, temperature 25 °C, adsorbent dose 0.1, 0.75, and 1.0 g L^−1^. (**b**) Effect of the initial pH. Conditions: adsorbent dose 0.75 g L^−1^, initial concentration of Hg^2+^ 1.0 mg L^−1^, stirring rate 270 rpm, time 24 h, temperature 25 °C, pH 3–10. (**c**) Effects of initial concentration of Hg^2+^. Conditions: pH 6.8, stirring rate 270 rpm, time 24 h, temperature 25 °C, adsorbent dose 0.75 g L^−1^, initial concentration of Hg^2+^ 1–100 mg L^−1^.

Figure 3a shows that Hg^2+^ removal efficiency increased from approximately 88% to 94% as the FAU-type X dosage was increased from 0.10 to 0.75 g L^−1^. This behavior can be attributed to the increase in the number of available adsorption sites, which enhances the probability of interaction between Hg^2+^ ions and the adsorbent surface. However, further increasing the adsorbent dosage did not produce significant improvement in Hg^2+^ removal efficiency, indicating that adsorption equilibrium had been reached under the experimental conditions. Therefore, a dosage of 0.75 g L^−1^ was selected as the optimum condition, as it provided high Hg^2+^ removal while minimizing adsorbent consumption.

Although increasing the adsorbent dosage increases the number of available adsorption sites, the effectiveness of these sites is strongly influenced by solution pH, which governs both the adsorbent surface charge and the speciation of Hg^2+^ in solution [46]. Therefore, the effect of pH on Hg^2+^ removal by FAU-type X was evaluated over the pH range of 3–10. As shown in Figure 3b, the highest Hg^2+^ removal efficiency (93.16%) was achieved at pH 6.8, corresponding to the natural pH of the mercury solution. In contrast, Hg^2+^ removal decreased to approximately 80% under acidic conditions and gradually declined to about 65% at alkaline pH values. To better understand this behavior, the predominant mercury species at different pH values were evaluated using speciation diagrams (Figure S5). Under acidic conditions (pH ≤ 6.0), the adsorption efficiency decreased due to the combined effect of surface protonation and the predominance of HgCl_2_(aq), which reduces the interaction of mercury species with the negatively charged adsorption sites of FAU-type X. In contrast, under alkaline conditions (pH 8–10), the reduced solubility of Hg(OH)_2_ limits the mobility and availability of mercury species in solution, thereby reducing adsorption efficiencies. At pH 6.8, HgClOH(aq) was identified as the predominant mercury species, coexisting with a lower concentration of HgCl_2_(aq) and Hg(OH)_2_(aq). The predominance of HgClOH(aq) likely favors adsorption due to its permanent dipole moment [47], which can enhance interactions with oxygen-containing functionalities and negatively charged sites present in the aluminosilicate framework of the zeolite. These findings suggest that the combined effects of mercury speciation and surface charge play a critical role in determining the adsorption performance of FAU-type X.

After identifying pH 6.8 as the most favorable condition for Hg^2+^ adsorption, the effect of the initial mercury concentration was evaluated over the range of 1–100 mg L^−1^ using FAU-type X as the adsorbent (Figure 3c). Hg^2+^ removal remained nearly constant at approximately 90% for concentrations between 1 and 20 mg L^−1^. However, adsorption efficiency decreased progressively with increasing Hg^2+^ concentration, reaching approximately 45% removal at an initial Hg^2+^ concentration of 100 mg L^−1^. This behavior indicates that, at low Hg^2+^ concentrations, the available adsorption sites in FAU-type X are sufficient to capture Hg^2+^ species. As the initial concentration increases, the number of mercury species in solution exceeds the available adsorption sites, leading to progressive saturation of the adsorbent surface and a consequent reduction in removal efficiency [48]. These results demonstrate the high affinity of FAU-type X toward Hg^2+^ at environmentally relevant concentrations.

### 2.4. Adsorption Isotherms

Adsorption equilibrium isotherms provide valuable information regarding the adsorption capacity of a material and the interaction between the adsorbate and the adsorbent surface [49]. Accordingly, the equilibrium adsorption data obtained for FAU-type X at different initial Hg^2+^ concentrations were fitted to the nonlinear Langmuir, Freundlich, and Sips isotherm models (Figure S6), and the corresponding parameters are summarized in Table 3. The Langmuir model assumes monolayer adsorption on a homogeneous surface containing energetically equivalent sites [50]. For FAU-type X, this model predicted a maximum adsorption capacity (*q*_m_) of 57.83 mg g^−1^. Moreover, the values obtained for the Langmuir affinity constant (*K*_L_) and the separation factor (*R*_L_) indicate a favorable adsorption process for Hg^2+^ onto the zeolite. In contrast, the Freundlich model describes adsorption on energetically heterogeneous surfaces [51]. The Freundlich constant (*K*_F_) obtained for FAU-type X was 11.05 mg g^−1^, whereas the adsorption intensity parameter (*n* = 2.375) indicates favorable adsorption according to the conventional interpretation of the Freundlich isotherm [52].

The Sips isotherm was further evaluated because it combines the characteristics of the Langmuir and the Freundlich models and accounts for adsorption on energetically heterogeneous surfaces [53]. The Sips model predicted a maximum adsorption capacity (q_MLF_) of 83.14 mg g^−1^, whereas the heterogeneity factor (M_LF_ < 1) confirmed the heterogeneous nature of the adsorption surface. Among the evaluated models, the Sips isotherm provided the best overall fit to the experimental data, exhibiting the highest correlation coefficient (R^2^ = 0.998) and the lowest average percentage error (APE = 4.504%). Nevertheless, the Langmuir model also provided an excellent description of the adsorption equilibrium (R^2^ = 0.997), suggesting that Hg^2+^ adsorption onto FAU-type X involves both homogeneous and heterogeneous adsorption domains. These observations indicate that adsorption occurs on a surface containing sites with different adsorption energies, where high-affinity sites are preferentially occupied at low Hg^2+^ concentrations, while lower-energy sites become progressively involved as concentration increases [54].

Although the Sips model provided the best fit, Langmuir adsorption capacity was selected for comparison with previously reported adsorbents because it remains the most widely reported parameter in the adsorption studies. As shown in Table 4, the adsorption capacity obtained for FAU-type X was higher than that reported for β-zeolite, Zeolite Y, Mordenite, and several modified zeolitic materials, despite employing lower adsorbent dosages. For comparison, a Langmuir adsorption capacity of 16.4 mg g^−1^ has been reported for commercial Zeolite 13X (FAU-type X), whereas the rice husk-derived FAU-type X investigated in this study exhibited a value of 57.8 mg g^−1^. However, because these values were obtained under different experimental conditions, particularly in terms of adsorbent dosage, initial Hg^2+^ concentration range, and solution pH, this comparison should be interpreted only as a literature benchmark rather than as evidence of direct performance superiority. Higher adsorption capacities have been reported for rice husk-derived NaX zeolite and its AgNP-modified counterpart [22]; however, these values were obtained under strongly acidic conditions (pH 2.5), further highlighting the importance of considering differences in experimental conditions when comparing adsorption performance across studies. Similarly, sulfur-modified zeolites, including sulfur-impregnated clinoptilolite and ZnS-zeolite NaA, exhibited substantially higher Hg^2+^ adsorption capacities. The enhanced performance of sulfur-containing adsorbents can be attributed to the strong affinity between Hg^2+^ species and sulfur functional groups, which generate highly specific adsorption sites [55]. Nevertheless, it is important to note that many of these studies required pH adjustment or chemical modification of the adsorbent surface. In contrast, FAU-type X achieved efficient Hg^2+^ removal at the natural pH of the aqueous solution without additional chemical treatment. These results highlight the potential applicability of FAU-type X for Hg^2+^ removal under realistic water treatment conditions.

### 2.5. Reuse of Materials and Possible Adsorption Interactions

**Figure 4:**
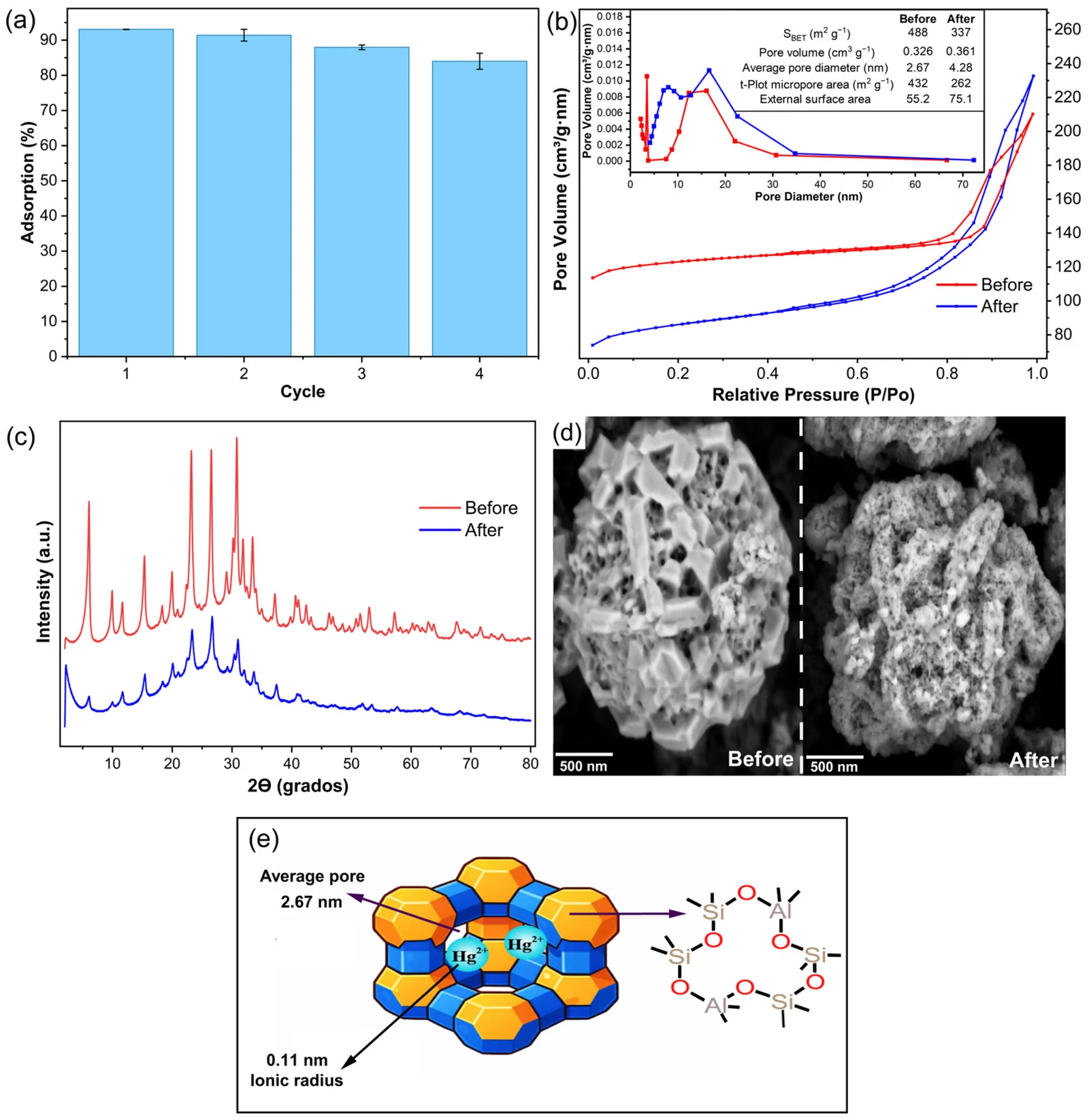
Changes in FAU-type X after four adsorption–desorption cycles: (**a**) Reuse. Conditions: pH 6.8, initial concentration of Hg^2+^ 1.0 mg L^−1^, stirring rate 270 rpm, adsorbent dose 0.75 g L^−1^, temperature 25 °C. (**b**) N_2_ adsorption–desorption isotherms with inset pore size distribution. (**c**) XRD before and after reuse. (**d**) SEM before and after reuse. (**e**) Contribution of the internal structure of the material to the adsorption process.

The reusability of FAU-type X for Hg^2+^ removal was evaluated through four consecutive adsorption–desorption cycles. As shown in Figure 4a, the adsorption efficiency gradually decreased upon reuse, although the material retained approximately 84% of its initial adsorption capacity after the fourth cycle. To elucidate the structural and physicochemical changes responsible for this decrease, complementary characterization analyses were performed. The nitrogen physisorption analysis (Figure 4b) revealed a reduction in nitrogen uptake upon repeated reuse, leading to a decrease in the specific surface area. In addition, the pore size distribution shifted toward larger pore diameters compared with the pristine material, as reflected by the reduced contribution of smaller pores and the increased contribution of pores above ~10 nm (Table 1 and Figure 4b inset). This shift was accompanied by only a modest increase in the average pore diameter, from 2.67 to 4.28 nm. Notably, this increase should not be interpreted as a physical enlargement of individual pores, but rather as a change in the relative weighting of different pore populations, these observations suggest that repeated adsorption–desorption cycles induced partial modification of the porous network, most likely reflecting a limited loss of microporosity, as indicated by the decrease in t-Plot micropore area from 432 to 262 m^2^ g^−1^, together with a slight increase in external surface area from 55.2 to 75.1 m^2^ g^−1^, resulting in an apparent increase in the relative contribution of mesopores to the measured pore size distribution. Furthermore, the XRD patterns (Figure 4c) show a progressive decrease in crystallinity after four adsorption cycles, indicating a partial loss of the long-range structural order of the zeolite framework. SEM observations (Figure 4d) further showed that although the particles preserved their overall spheroidal morphology, the surface became progressively more irregular and porous, suggesting slight surface erosion during repeated use. Overall, these findings are consistent with predominant Hg^2+^ retention within the zeolite’s porous structure, favored by the dimensional compatibility between the average pore size and the Hg^2+^ (Figure 4e).

**Figure 5:**
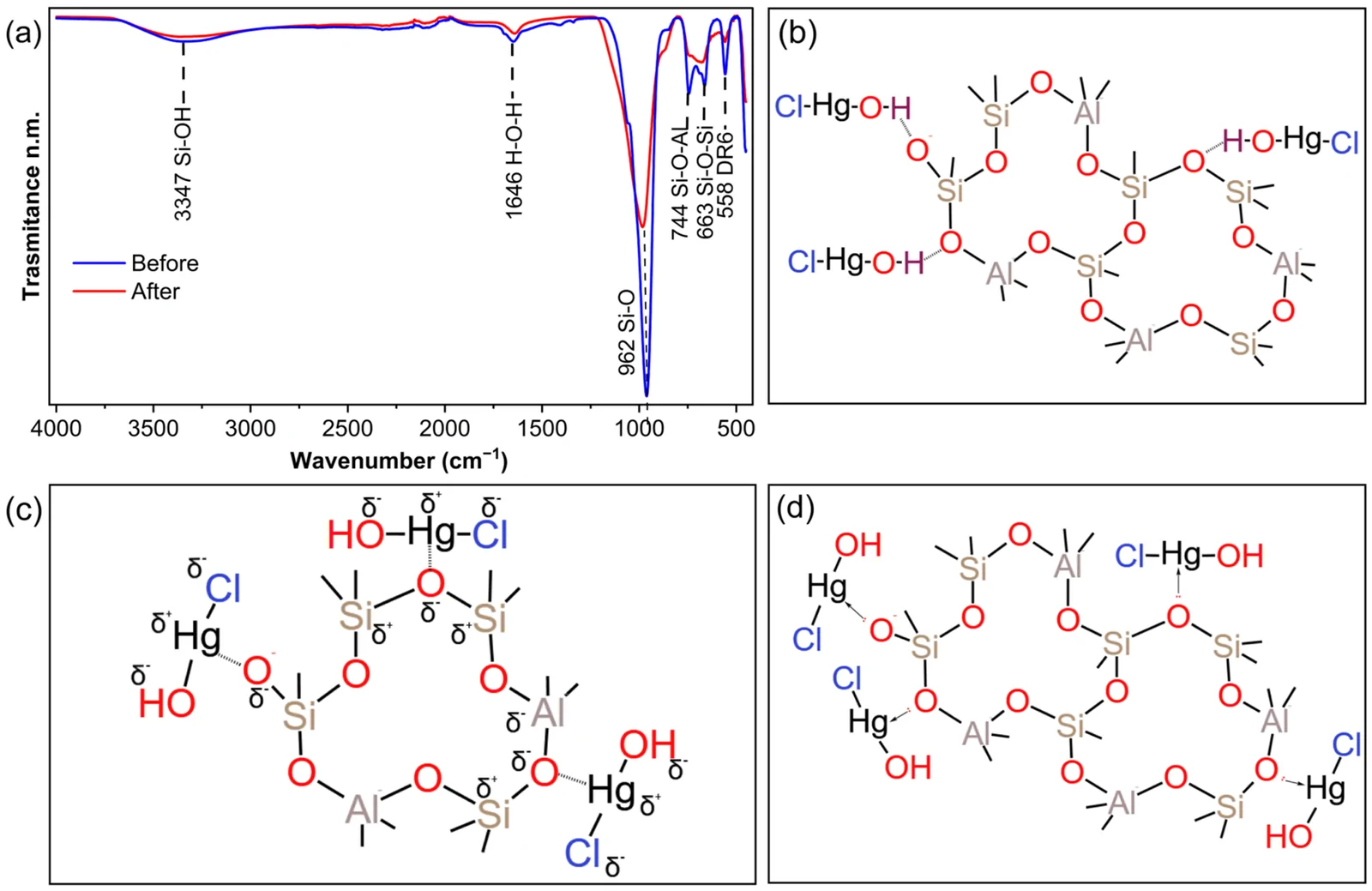
Proposed mechanisms of Hg^2+^ adsorption onto FAU-type X: (**a**) FTIR, (**b**) hydrogen-bonding interactions, (**c**) dipole interactions, and (**d**) acid–base interactions of the FAU-type X groups, such as Si–O^−^, Si–O–Si, and Si–O–Al, with HgClOH.

To further elucidate the interactions involved in Hg^2+^ removal, FTIR analyses were conducted before and after adsorption (Figure 5a). Following Hg^2+^ adsorption, a decrease in the intensity of the band at 962 cm^−1^, assigned to Si–O vibrations associated with silanol groups, was observed together with the partial merging of the bands located at 744 and 663 cm^−1^, corresponding to Si–O–Al and Si–O–Si vibrations, respectively. These spectral changes suggest that oxygen-containing surface functionalities participate in the adsorption process. Under the experimental conditions (pH 6.8), deprotonated silanol groups (Si–O^−^), together with oxygen atoms associated with Si–O–Si and Si–O–Al bridges, may interact with Hg^2+^ species through electrostatic attraction and ion-exchange mechanisms (Figure 5b). In particular, the higher electron density associated with Si–O–Al linkages may favor stronger interactions with mercury species due to the presence of framework negative charges generated by aluminum incorporation into the zeolite structure. Moreover, HgClOH, the predominant mercury specie under the experimental conditions, possesses a permanent dipole moment, which may promote dipole-surface interactions with the oxygen-containing functionalities present in the aluminosilicate framework (Figure 5c). In addition, weak Lewis acid–base interactions between Hg species and accessible oxygen atoms of the zeolite framework may further contribute to the Hg^2+^ adsorption mechanism (Figure 5d). Overall, these findings reinforce the thermodynamic interpretation presented in Section 2.2, indicating that Hg^2+^ adsorption on FAU-type X is a favorable and spontaneous process. Although the proposed mechanism explains Hg^2+^ retention by FAU-type X under controlled conditions, its effectiveness in natural water must also be examined.

### 2.6. Hg^2+^ Removal from River Water with Ecotoxicological and Microbiological Evaluation

**Figure 6:**
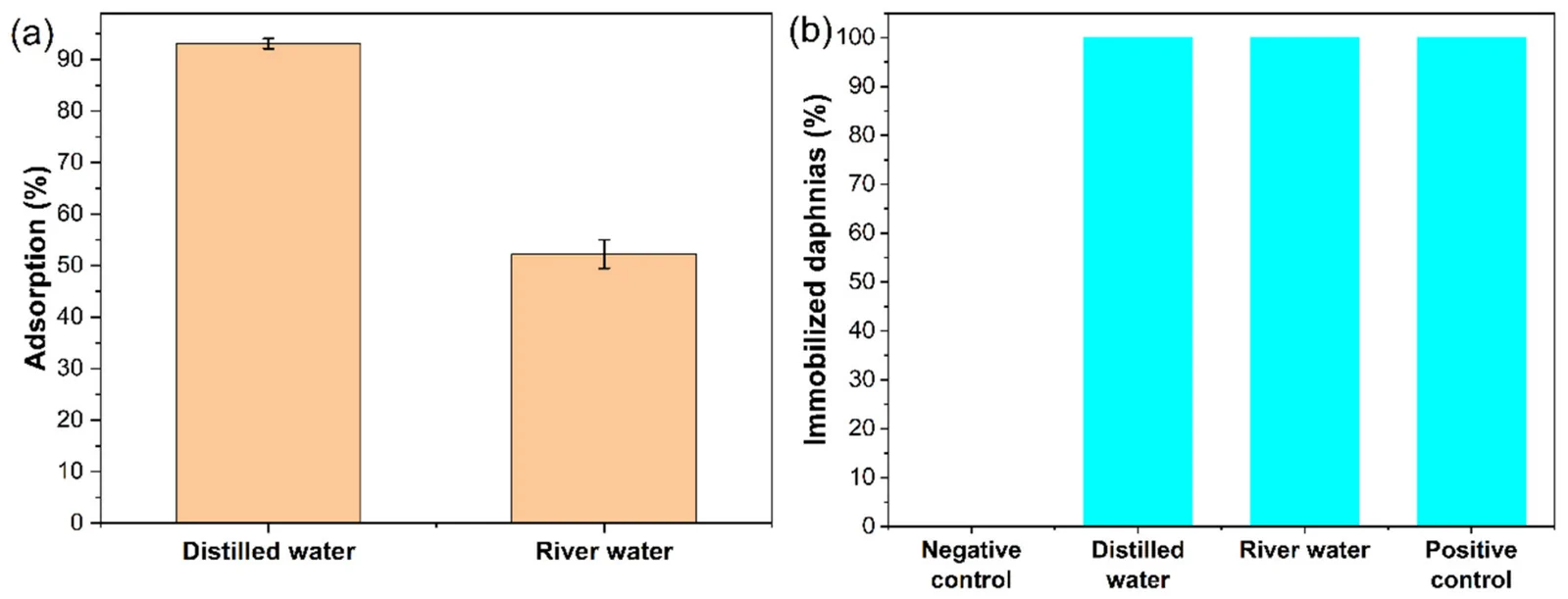
Behavior of FAU-type X in the removal of Hg^2+^ from river and distilled water: (**a**) adsorption and (**b**) ecotoxicological assessment after treatment with FAU-type X. Conditions: initial concentration Hg^2+^ 1.0 mg L^−1^, pH 6.8, stirring rate 270 rpm, adsorbent dose 0.75 g L^−1^, time 24 h, temperature 25 °C.

To assess the applicability of FAU-type X under environmentally relevant conditions, Hg^2+^ adsorption experiments were conducted using river water samples, and the results are presented in Figure 6a. Compared with distilled water, FAU-type X exhibited a lower Hg^2+^ removal efficiency in river water, achieving only 52%. This decrease is likely associated with the presence of competitive ions, including Al, Fe, Ni, Cu, Zn, Cd, and Sn, identified by X-ray fluorescence analysis (Table S3), which may compete with Hg^2+^ for the available adsorption sites on FAU-type X [62]. A similar challenge arises in other complex aqueous matrices, such as seawater, where high ionic strength and competing cations can likewise hinder Hg^2+^ uptake. To address this limitation, other authors have modified commercial FAU-type X (Zeolite 13X) through sulfide functionalization, achieving nearly complete Hg^2+^ removal (99%) even under such demanding conditions [63]. This highlights surface modification as a promising strategy to enhance the selectivity of FAU-type X toward Hg^2+^ in complex real-water matrices.

For FAU-type X in this work, the residual Hg^2+^ concentration after 24 h of treatment increased from 0.065 mg L^−1^ in distilled water to 0.48 mg L^−1^ in river water. Considering the residual Hg^2+^ concentrations remaining after treatment and their potential environmental implications, an ecotoxicological assay was conducted with *Daphnia magna*. As shown in Figure 6b, complete immobilization of *Daphnia magna* was observed in both treated matrices. Mercury is known to induce toxicity through multiple mechanisms, including disruption of cellular macromolecules, inhibition of essential enzymatic functions, and impairment of neurological functions [64]. Furthermore, probit analysis performed over the Hg^2+^ concentration range of 0.0031–0.075 mg L^−1^ yielded an EC_50_ value of 0.0220 mg L^−1^ (Figure S7). Since the residual Hg^2+^ concentrations remaining after adsorption exceeded this threshold, the complete immobilization observed in both matrices can be attributed to the persistence of toxic mercury concentration after treatment. These findings demonstrate that high adsorption efficiencies do not necessarily guarantee environmental safety and emphasize the importance of integrating ecotoxicological assessments into adsorption studies.

In addition to the ecotoxicological assessment, microbiological analyses involving *E. coli* and total coliforms were conducted using river water samples. As shown in Table S5, untreated river water samples contained 33 CFU 100 mL^−1^ of *E. coli* and 800 CFU 100 mL^−1^ of total coliforms. Significant reductions in total coliform counts and complete removal of *E. coli* were observed in river water treated with FAU-type X in the absence of Hg^2+^. Moreover, no detectable microorganisms were observed after treatment of Hg^2+^-contaminated river water with FAU-type X. The absence of detectable microorganisms in Hg^2+^-containing samples may be partially attributed to the well-documented toxicity of Hg^2+^ toward bacterial cells, even at relatively low concentrations [65]. However, the reduction in bacterial counts observed in the absence of Hg^2+^ suggests that FAU-type X itself contributes to microbial removal through adsorption-driven processes rather than exhibiting an intrinsic antibacterial activity. Considering the negatively charged surface of *E. coli* cells [66] and the positive surface charge of FAU-type X, as indicated by its PZC value (Table 1), electrostatic interactions may facilitate bacterial retention on the adsorbent surface and within its porous structure.

Overall, although FAU-type X exhibited promising Hg^2+^ adsorption performance, residual mercury concentrations remaining after treatment still caused adverse effects on *Daphnia magna* and likely contributed to reduce bacterial populations. These findings demonstrate that adsorption efficiency alone is insufficient to assess the environmental safety of treated water and highlight the importance of incorporating ecotoxicological and microbiological evaluations into the development of water treatment technologies. Therefore, additional treatment strategies are required before the treated water is considered suitable for environmental discharge or agricultural reuse.

### 2.7. Evaluation of Phytotoxicity in Lactuca sativa

**Figure 7:**
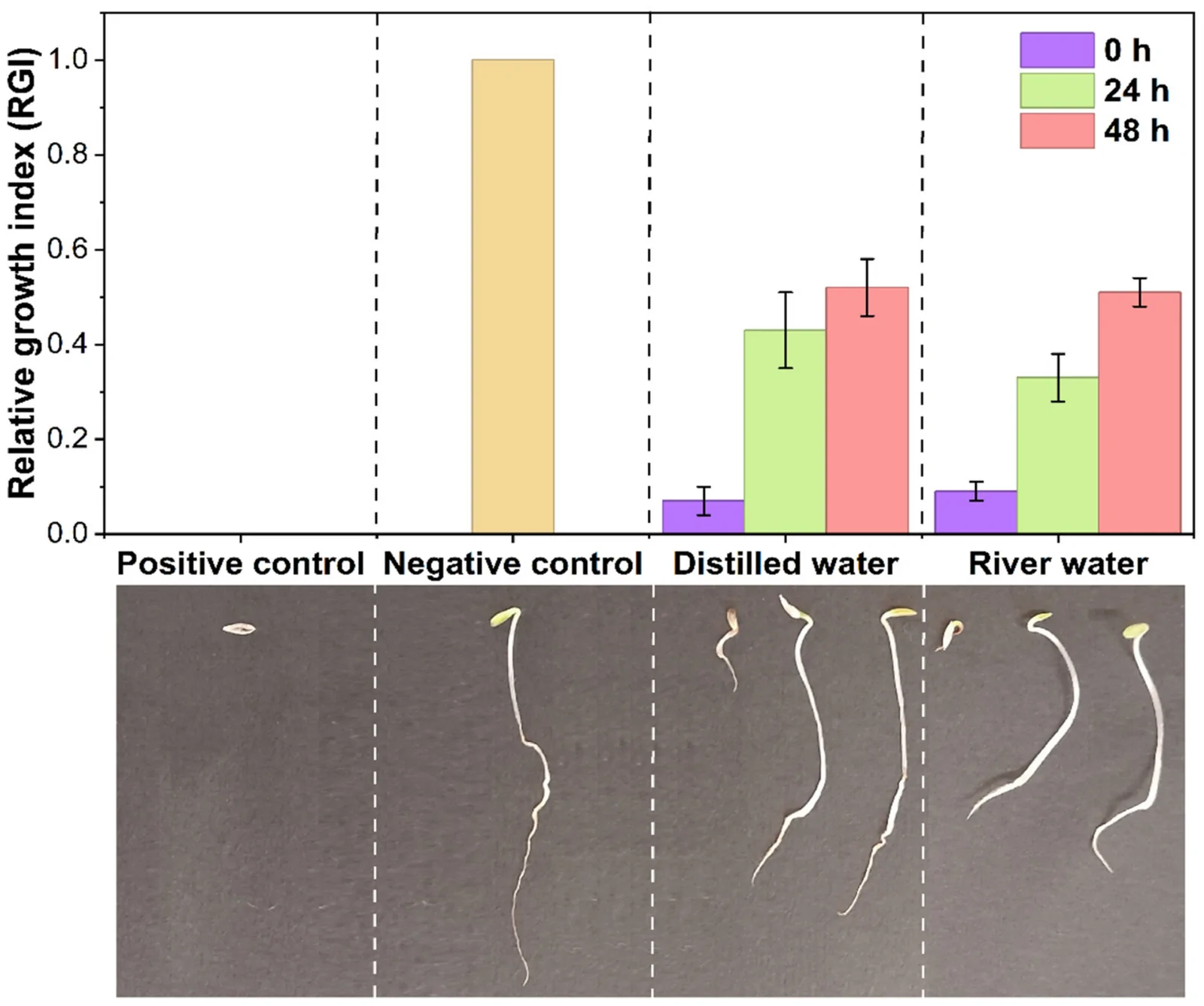
Relative growth index in *Lactuca sativa*. Conditions: 22 °C, darkness, Hg^2+^ 1.0 mg L^−1^, adsorbent dose 0.75 g L^−1^, pH 6.8.

The environmental impact of the treated water was further evaluated through phytotoxicity assays using romaine lettuce (*Lactuca sativa*) seeds. As shown in Figure 7, the positive control containing ZnSO_4_ caused complete inhibition of seedling growth, likely due to a high zinc concentration, which has been reported to induce oxidative stress and suppress plant development [67]. In contrast, seeds cultivated in distilled water (negative control) exhibited a relative growth index (RGI) value of 1.0, confirming that the experimental conditions supported normal seed germination and seedling development. Compared with the negative control, both aqueous matrices spiked with Hg^2+^ at time zero exhibited marked reductions in seed germination and growth, with RGI values below 0.1. After 24 h of FAU-type X treatment, the RGI values increased, and after 48 h, they reached approximately 0.5, indicating moderate inhibition of seedling elongation according to the RGI classification criteria (Table S6). The similar RGI values observed for both treated matrices suggest that the phytotoxic response was predominantly governed by the residual Hg^2+^ concentrations remaining after adsorption. The observed phytotoxicity can be attributed to the strong affinity of mercury species for sulfhydryl (–SH) groups present in proteins and enzymes involved in seed germination and early plant development. The formation of stable Hg–S complexes may disrupt essential metabolic pathways, impair enzymatic activity, and interfere with embryonic development processes [68]. In addition, the slightly lower plant growth observed in river water may also be associated with the presence of competitive metal ions, particularly aluminum, identified by X-ray fluorescence analysis (Table S3), which has been reported to inhibit root elongation and affect plant growth [69]. Overall, these findings highlight the need for additional treatment strategies to further decrease residual mercury concentrations before considering the treated water suitable for agricultural reuse. Furthermore, future studies involving crop species with different sensitivities to mercury toxicity are necessary to establish safe reuse criteria for treated water.

## 3. Materials and Methods

### 3.1. Reagents

Hg^2+^ was prepared from HgCl_2_ supplied by Panreac (Darmstadt, Germany). Aluminum sulfate octadecahydrate (Al_2_(SO_4_)_3_·18H_2_O) was obtained from Alpha Chemika (Bombay, India), while zinc sulfate (ZnSO_4_) was provided by Merck (Darmstadt, Germany). Additional reagents, including sodium hydroxide (NaOH), cetyltrimethylammonium bromide (CTAB), and nitric acid (69%) were purchased from Panreac (Darmstadt, Germany), whereas hydrochloric acid (37%) was sourced from Honeywell (Seelze, Germany), and sodium hypochlorite (NaClO) was provided by Clorquímicos (Ibagué, Colombia). For preparing the saline solution (Table S7) used in ecotoxicity assays, sodium bicarbonate (NaHCO_3_), calcium chloride dihydrate (CaCl_2_·2H_2_O), magnesium sulfate heptahydrate (MgSO_4_·7H_2_O), and potassium chloride (KCl) were obtained from Microbiotests (Gent, Belgium). Additionally, *Daphnia magna*, Chromocult test kits, and organic lettuce were acquired from Microbiotests (Gent, Belgium), Sartorius (Göttingen, Germany), and Anasac (Bogotá, Colombia), respectively. Chromocult coliform agar (Merck, Darmstadt, Germany) was prepared according to the supplier’s guidelines [70]. The river water sample was collected from Cuenca Alta, Pradera, Curillo (Caquetá, Colombia), and rice husk (RH) was provided by agro-industries located in Espinal (Tolima, Colombia).

### 3.2. Preparation of Adsorbents

#### 3.2.1. Faujasite-Type Y

Silica was extracted from rice husk using a sol–gel method adapted from previous studies [71,72]. Briefly, 80 g of rice husk was washed with distilled water and dried at room temperature. The dried material was then acid-treated with 0.1 M HCl, followed by alkaline digestion with 5 M NaOH under continuous stirring at 60 °C for 6 h. After standing and filtration, the filtrate was neutralized with 20% nitric acid to pH 7, forming a gel aged for three days. The gel was washed with 13% sodium hypochlorite until a white solid was obtained, aged for 24 h, dried at 80 °C, and subsequently ground to obtain the silica precursor. The silica was refluxed with 2.3 M NaOH for 4 h to produce sodium silicate. In parallel, Al(OH)_3_ was prepared by mixing 2.3 M NaOH with 2.3 M Al_2_(SO_4_)_3_·18H_2_O.

Faujasite-type Y was synthesized following previous studies, with some modifications [73,74]. Sodium silicate was combined with aluminum hydroxide under stirring at 45 °C for 2 h and aged for 24 h in darkness. Hydrothermal synthesis was then performed at 100 °C for 48 h, and the resulting solid was filtered and washed until the filtrate reached pH 10, then dried and ground to obtain the final FAU-type Y material.

#### 3.2.2. Faujasite-Type X

Silica was obtained from rice husk via alkaline extraction [17,75]. A total of 60 g of rice husk was washed, dried, treated with 0.1 M HCl, and calcined at 700 °C for 2 h. The resulting rice husk ash (RHA) was dissolved in 2 M NaOH (1 g/10 mL) under stirring at 60 °C and 150 rpm for 4 h, and the silicate was separated by filtration and centrifugation. Al(OH)_3_ was prepared in parallel by mixing 2 M NaOH with 2 M Al_2_(SO_4_)_3_·18H_2_O.

Faujasite-type X was synthesized following previously reported procedures with some modifications [76,77,78]. The silicate solution was added dropwise to the aluminum hydroxide precursor under continuous stirring, followed by the addition of 0.52 mmol of CTAB. The gel was left in the dark for 20 days. Finally, the material was hydrothermally treated at 90 °C for 12 h, then filtered, dried, ground, and calcined at 300 °C for 5 h to obtain the final FAU-type X material.

### 3.3. Characterization Techniques

The zeolites were analyzed by X-ray diffraction (XRD) employing a MiniFlex 600 diffractometer with Cu Kα radiation (Tokio, Japan). Bragg’s equation enables the calculation of the interplanar spacing (d_hkl_) (Text S1), and phase identification was carried out using the PDF-4 database (ICDD—International Center for Diffraction Data). Fourier-transform infrared (FT-IR) spectra were obtained within the 4000–400 cm^−1^ interval using a Spectrum Two spectrometer (Seer Green, UK). Even changes in the spectra before and after Hg^2+^ uptake were analyzed to propose a plausible adsorption mechanism. Surface morphology and composition were evaluated through scanning electron microscopy (SEM) combined with energy-dispersive X-ray spectroscopy (EDS) using a Thermo Fisher Scientific Scios 2 LoVac instrument (Brno, Czech Republic). The point of zero charge (pH_PZC_) was established following the solid addition method [79]. Nitrogen adsorption–desorption analyses were performed on a 3Flex Micromeritics instrument (Norcross, GA, USA). The specific surface area was estimated using the Brunauer–Emmett–Teller (BET) approach, the average pore diameter was determined by the BET results (4V/A by BET), while the pore size distribution was derived from the desorption branch applying the Barrett–Joyner–Halenda (BJH) model, and the pore volume was calculated at P/Po = 0.991. In addition, the elemental composition of river water was determined by X-ray fluorescence (XRF) with a Nex QC+ system (Galveston, TX, USA).

### 3.4. Analysis of Adsorption, Kinetics and Thermodynamics

FAU-type Y and FAU-type X were evaluated via adsorption experiments conducted in 50 mL flasks on a mechanical shaker at 270 rpm for contact times ranging from 5 to 1440 min, using an initial Hg^2+^ concentration of 1 mg L^−1^ and an adsorbent dosage of 1 g L^−1^. Furthermore, the adsorption kinetics of Hg^2+^ onto FAU-type Y and FAU-type X were investigated at three temperatures (25, 45, and 65 °C). The adsorption experiments were conducted using the same pollutant concentration and adsorbent dose for 700 min, corresponding to the equilibrium time. All experiments were conducted in hermetically sealed systems to prevent evaporation losses.

The kinetic data obtained at each temperature were fitted using the nonlinear pseudo-first-order and pseudo-second-order models (Text S2 and Text S3, respectively). For the thermodynamic analysis in the range of temperature 25–65 °C, the changes in standard Gibbs free energy (ΔG°), enthalpy (ΔH°), and entropy (ΔS°) were determined (Text S4). Finally, the adsorbent with the best overall performance was selected for subsequent studies considering its adsorption kinetics, reflected by higher correlation coefficients (R^2^) and lower average percentage errors (APE, %) (Text S5), together with its more favorable thermodynamic properties in terms of spontaneity and energy requirements.

### 3.5. Operational Parameters and Isotherms

The selected material was further used to evaluate the influence of key operating variables, such as adsorbent dosage (0.1–1.0 g L^−1^), solution pH (3–10), and the effect of the initial Hg^2+^ concentration (1–100 mg L^−1^). Consequently, the reported pollutant concentration results were used to estimate the maximum adsorption capacity of Hg^2+^ on the zeolite by fitting the nonlinear Langmuir, Freundlich, and Sips isotherm models (Text S6, Text S7, and Text S8, respectively). The most suitable model describing the adsorption behavior was selected considering the correlation coefficient (R^2^) and the average percentage error (APE, %).

### 3.6. Reuse Cycles of the Material and Proposed Adsorption Mechanism

To evaluate the adsorbent’s reusability, sequential adsorption–desorption cycles were performed 4 times. Each cycle began with an adsorption step conducted under the previously established optimal conditions, followed by a regeneration stage through desorption. For desorption, the spent adsorbent was first air-dried at room temperature and then treated with 50 mL of a 1 M thiourea solution for 2 h under the same conditions. After regeneration, the FAU-type X material was reused in new Hg^2+^ adsorption tests.

### 3.7. Phytotoxicity

Seed germination tests were conducted with romaine lettuce (*Lactuca sativa*). Before testing, the seeds were disinfected by immersing them in a 1.5% sodium hypochlorite solution for 2 min with intermittent shaking, followed by 2 washes with sterile distilled water [80]. To minimize microbial contamination, the empty Petri dishes were additionally exposed to ultraviolet radiation at full intensity for 30 min. For the experimental controls, zinc sulfate at 400 mg L^−1^ was prepared as the positive reference solution, whereas the negative control consisted of distilled water only.

Each Petri dish was filled with 4 mL of either distilled water or river water samples collected at 0, 24, and 48 h of treatment. Twenty seeds were placed in each dish, and all conditions were prepared in triplicate. The dishes were then incubated at 22 °C under complete darkness. Seedling development proceeded for at least five days before evaluation, and the relative growth index (RGI) was calculated (Text S9).

### 3.8. Ecotoxicity and Microbiological Tests

The bioassay was performed in accordance with the Standard Operating Procedure of the Laboratory for Environmental Toxicology and Aquatic Ecology at Ghent University [81]. The toxicity assay employed neonates obtained from ephippia, which were activated four days in advance. To trigger hatching, the dormant eggs were maintained at 22 °C under continuous illumination until viable organisms emerged for testing. On the day of exposure, freshly hatched neonates were given Spirulina 3 h before testing to ensure adequate nutritional status. The assay medium was prepared using a defined saline solution (Table S7). For the experimental design, multi-well plates were arranged with four replicates for each tested concentration, as well as for the positive control (K_2_Cr_2_O_7_) and the negative control (distilled water). Each replicate received five neonates placed into 10 mL of test solution, maintaining a minimum ratio of 2 mL per organism. Following inoculation, the plates were sealed with Parafilm, covered, and incubated in darkness at 22 °C. Organism responses were subsequently assessed after 24 h of exposure. In addition, the EC_50_ was determined by evaluating the immobilization of *Daphnia magna* over the Hg^2+^ concentration range of 0.0031–0.075 mg L^−1^ in saline solution, with a 24 h exposure time, and EC_50_ values were calculated using Probit analysis with freely available statistical software.

For microbiological enumeration, sterile dehydrated nutrient pads were employed in combination with the membrane filtration technique using 0.45 µm filters. In this approach, a 100 mL portion of each water sample was passed through the membrane under vacuum, after which the filter was carefully transferred onto the hydrated pad inside a sterile Petri dish. The pads were pre-wetted with 3.0–3.5 mL of sterile water to create adequate moisture for bacterial growth. The assembled plates were then incubated at 37 °C for 48 h in the absence of light, and three replicate cultures were prepared for each sample. Following incubation, total coliforms were recognized by the appearance of pink to red colonies, while *Escherichia coli* was distinguished by the formation of dark blue to violet colonies according to the chromogenic reaction of the culture medium.

### 3.9. Analytical Techniques

Hg^2+^ concentrations were determined with a Lumex RA-915 LAB mercury analyzer (Mission, BC, Canada), employing atomic absorption spectrometry at 253.7 nm with Zeeman-effect background correction. Quantitative analysis was based on a certified Hg(NO_3_)_2_ reference standard, yielding a calibration curve with an R^2^ of 0.9990, and a method quantification limit of 0.0036 µg mL^−1^.

## 4. Conclusions

The characterization analyses confirmed the successful synthesis of FAU-type X and FAU-type Y, which exhibited the characteristic structural features of faujasite zeolites. Although both materials exhibited comparable crystalline frameworks, FAU-type X showed substantially superior Hg^2+^ adsorption performance, indicating that the adsorption process performance was governed primarily by the accessibility and chemical nature of the adsorption sites rather than by specific surface area alone. The enhanced adsorption performance of FAU-type X was attributed to its larger pore diameter, higher pore volume, more heterogeneous morphology, and greater abundance of Si–OH and Si–O–Al active sites. Furthermore, the faster adsorption kinetics and more favorable thermodynamic parameters observed for FAU-type X suggest that Hg^2+^ adsorption occurs more readily and with lower energetic requirements than FAU-type Y, highlighting the potential of rice husk-derived Faujasite X as a promising adsorbent for mercury removal from aqueous systems.

Under the optimized conditions (pH 6.8, 24 h, and a zeolite dose of 0.75 g L^−1^), FAU-type X exhibited high Hg^2+^ removal efficiency. The equilibrium data were best described by the Sips isotherm model, indicating adsorption on an energetically heterogeneous surface with a q_MLF_ of 83.14 mg g^−1^. Reusability studies revealed that, despite partial structural rearrangement and loss of crystallinity after four adsorption–desorption cycles, FAU-type X retained approximately 84% of its initial adsorption capacity. Spectroscopic and thermodynamic analyses suggest that Hg^2+^ retention occurs predominantly through electrostatic attraction, ion-exchange processes, dipole interactions, and weak Lewis acid–base interactions involving oxygen-containing functionalities present in the zeolite framework.

Although FAU-type X exhibited promising Hg^2+^ removal performance, its adsorption efficiency decreased (52%) in river water due to competition from coexisting ions. In addition, FAU-type X contributed to the reduction in microbial population, likely through adsorption-driven mechanisms. However, the residual Hg^2+^ concentrations remaining after treatment still induced adverse effects on *Daphnia magna* and inhibited the growth of *Lactuca sativa* seedlings (RGI = 0.5). These findings demonstrate that adsorption efficiency alone is insufficient to ensure the environmental safety of treated water and highlight the importance of integrating ecotoxicological and phytotoxicological assessments into the development of water treatment technologies. Therefore, additional treatment strategies are required before the treated water is considered suitable for environmental discharge or agricultural reuse.

## Figures and Tables

**Table 1:** Surface characteristics of adsorbents.

Chemical and Surface Analysis	Characteristic	Adsorbent	
		FAU-Type Y	FAU-Type X
FTIR			
Wavenumber (cm^−1^)	Functional group		
3347	Si-OH	Present	Increased
1646	H-O-H	Present	Increased
962	Si-O	Present	Increased
744	Si-O-Al	Present	Increased
663	Si-O-Si	Present	Increased
558	DR6	Present	Increased
Surface charge	PZC	9.08	9.27
Nitrogen physisorption analysis	S_BET_ (m^2^ g^−1^)	679	488
	Pore volume (cm^3^ g^−1^)	0.267	0.326
	Average pore diameter (nm)	1.57	2.67

**Table 2:** Thermodynamic parameters in the removal of Hg^2+^ using zeolites. Conditions: adsorbent dose 1.0 g L^−1^, initial concentration of Hg^2+^ 1 mg L^−1^, pH 6.8, temperature 25, 45, and 65 °C.

Thermodynamic Parameters				
Adsorbent	Temperature (°C)	∆H (kJ mol^−1^)	∆G (kJ mol^−1^)	∆S (J mol^−1^ K^−1^)
FAU-type Y	25	18.91 ± 0.36	−3.48 ± 0.18	75.12 ± 3.52
	45		−4.99 ± 0.36	
	65		−6.50 ± 0.23	
FAU-type X	25	5.32 ± 0.29	−6.43 ± 0.67	39.40 ± 0.93
	45		−7.22 ± 0.29	
	65		−8.01 ± 0.65	

**Table 3:** Adsorption parameters for Langmuir and Freundlich isotherms. Conditions: pH 6.8, stirring rate 270 rpm, time 24 h, adsorbent dose 0.75 g L^−1^, temperature 25 °C, initial concentration Hg^2+^ 1–100 mg L^−1^.

Isotherm	Parameters	Values Obtained
Langmuir	q_m_ (mg g^−1^)	57.83
	K_L_ (L mg^−1^)	0.187
	RL	0.889
	R^2^	0.968
	APE (%)	5.685
Freundlich	K_F_ (mg g^−1^)	11.05
	n	2.375
	R^2^	0.984
	APE (%)	7.772
Sips	q_MLF_ (mg g^−1^)	83.14
	M_LF_	0.551
	R^2^	0.987
	APE (%)	4.504

**Table 4:** Comparison of the adsorption capacity of FAU-type X with other adsorbents.

Adsorbent	q_m_ (mg g^−1^)	S_BET_	pH	Adsorbent Dose (g L^−1^)	Initial Concentration Hg^2+^ (mg L^−1^)	Reference
β-zeolite	1.94	575	6	7	5	[37]
FAU-type Y	1.61	660	6	6		
Mordenite	0.76	250	7	4		
zeolites@S-0	4.40	18	4	5	200	[56]
β-zeolite	9.72	619	7	5	5–100	[57]
Commercial FAU-type X	16.4	635	5–6	100	13–575	[58]
NH_3_·H_2_O-zeolite 4A	53.6	28	6	2	10	[59]
FAU-type X	57.8	488	6.8	0.75	1–100	This study
Synthetic Zeolite NaX	137	6	2.5	10	25–400	[22]
Synthetic Zeolite NaX-modified AgNPs	286					
Sulfur-impregnated natural zeolite clinoptilolite	204	12	2	10	100	[60]
ZnS-zeolite NaA	553	No report	5	0.5	470	[61]

## Data Availability

The original contributions presented in this study are included in the article/Supplementary Materials. Further inquiries can be directed to the corresponding authors.

## Supplementary Materials

The following supporting information can be downloaded at: https://www.mdpi.com/article/10.3390/molecules31173101/s1. Text S1: Interplanar Spacing. Text S2: Description of the pseudo-first-order kinetic model. Text S3: Description of the pseudo-second-order kinetic model. Text S4: Determination of thermodynamic parameters. Text S5: Average percentage error (APE). Text S6: Description of the Langmuir isotherm. Text S7: Description of the Freundlich isotherm. Text S8: Description of the SIPS isotherm. Text S9: Relative growth index (RGI). Table S1: Elemental composition of adsorbents by energy-dispersive X-ray spectroscopy (EDS). Table S2: Interplanar spacing in FAU-type Y and FAU-type X. Table S3: X-ray fluorescence analysis of real-water samples (mass %). Table S4: Elemental composition of adsorbents by X-ray fluorescence analysis. Table S5: Microbiological analysis in Caquetá River. Conditions: Temperature 37 °C, adsorbed dose 0.75 g L^−1^, stirring rate 220 rpm. Table S6: Classification of RGI values. Table S7: Composition of saline solution. Figure S1: FTIR analysis of zeolites FAU-type Y and FAU-type X. Figure S2: Point of charge zero. Conditions: NaCl 0.1 M. adsorbent dose 1.0 g L^−1^. stirring rate 200 rpm, temperature 25 °C. Figure S3: N_2_ adsorption–desorption isotherms with inset pore size distribution for: (a) FAU-type Y and (b) FAU-type X. Figure S4: ln Kc vs. 1/T to obtain the thermodynamic parameters in Hg^2+^ adsorption. Conditions: Hg^2+^ concentration 1.0 mg L^−1^, dose 1.0 g L^−1^, pH 6.8, time 12 h., temperature 25–65 °C. Figure S5: Distribution of Hg^2+^ species diagram using the Visual MINTEQ program. Conditions: [Hg] = 0.90 mg L^−1^, [HCl] = 0.001 mol L^−1^. Figure S6: Langmuir. Freundlich and SIPS isotherms. Conditions: pH 6.8, stirring rate 270 rpm, time 24 h, adsorbent dose 0.75 g L^−1^, temperature 25 °C. initial concentration Hg^2+^ 1–100 mg L^−1^. Figure S7: Ecotoxicological assessment: (a) Immobility of *Daphnia magna*. (b) Probit at 24 h. Conditions: pH 6.5, temperature 22 °C, distilled water with saline solution (Table S7). References [82,83,84,85,86,87,88,89,90,91,92,93,94,95,96] are cited in the Supplementary Materials.

## Abbreviations

The following abbreviations are used in this manuscript:

RH	Rice husk
RHA	Rice husk ash
FAU	Faujasite
PFO	Pseudo-first-order
PSO	Pseudo-second-order
APE	Average percentage error
XRD	X-ray diffraction
XRF	X-ray fluorescence
EDS	Energy-dispersive spectroscopy
RGI	Relative growth index
PZC	Point of zero charge
