# Supplementary material for: Sustainable Synthesis of Faujasite-Type Zeolites Synthesized from Rice Husk for Hg2+ Removal from Aqueous Solutions: Adsorption Performance, Mechanistic Insights, and Environmental Safety Assessment
Source: Molecules. 2026 Sep 4;31(17):3101. doi: 10.3390/molecules31173101 (PMC13567045; doi:10.3390/molecules31173101)
Supplement: Supplementary file 1 [file molecules-31-03101-s001.zip › molecules-4449012-supplementary.pdf]

SUPPLEMENTARY MATERIAL

Sustainable Synthesis of Faujasite-types Zeolite synthesized from rice husk for Hg^{2+} Removal from Aqueous Solutions: Adsorption Performance, Mechanistic Insights, and Environmental Safety Assessment

Naren Bocanegra [1], Marcela Paredes-Laverde [2], Nancy Acelas [3,*], Ximena Carolina Pulido [1], Luis Rodríguez [1] and César Jaramillo-Páez [1,*]

1 Grupo de Investigación en Química Aplicada a Procesos Ecológicos (QUAPE-UT), Facultad de Ciencias, Universidad del Tolima, Ibagué, Colombia; nbocanegram@ut.edu.co (N.B.); xpulido@ut.edu.co (X.C.P.); lfrodriguezh@ut.edu.co (L.R.)
2 Grupo de Investigación Navarra Medicina, Facultad de Ciencias de la Salud, Fundación Universitaria Navarra-Uninavarra, Neiva, Colombia; maparedesla@gmail.com
3 Grupo de Investigación Materiales con Impacto (Mat&mpac), Facultad de Ingenierías, Universidad de Medellín, Medellín, Colombia
* Correspondence: nyacelas@udemedellin.edu.co (N.A.); cajaramillopa@ut.edu.co (C.J.-P.)

Text S1. Interplanar Spacing

Interplanar spacing (d_{hkl}) refers to the distance between two parallel atomic planes, and its values were determined using Bragg's equation [1]:

$$d_{hkl} = \frac{n\lambda}{2\,sin\,\theta} \quad (S1)$$

where d is the distance between two parallel atomic planes, n is a positive whole number, and λ is the wavelength of the incident electromagnetic wave and θ is the angle of reflection.

Text S2. Description of the pseudo-first-order kinetic model

The Lagergren pseudo-first-order model relates the adsorption rate to the availability of sites on the adsorbent, generally associated with physisorption [2], and is expressed as:

$$q_t = q_e(1 - e^{-k_1 t}) \quad (S2)$$

where q_t represents the amount adsorbed at time t (mg·g^{-1}), q_e the adsorption capacity at equilibrium (mg·g^{-1}), and k_1 the rate constant (min^{-1}) [3].

The determination of the amount adsorbed over time or at equilibrium $q_{t\,or\,eq}$ (mg g^{-1}) was calculated using:

$$q_{t\,ot\,eq} = \frac{C_0 - C_{t\,or\,eq}}{m} * V \quad (S3)$$

where C_0 is the initial concentration (mg L^{-1}); $C_{t\,or\,eq}$ is the concentration at time *t* or at equilibrium *eq* (mg L^{-1}); *m* is the mass of adsorbent used (g); *V* is the volume of the aqueous solution (L).

Text S3. Description of the pseudo-second-order kinetic model

The pseudo-second-order model characterizes adsorption controlled by chemical interactions between the adsorbate and surface functional groups [4], and it is expressed as:

$$q_t = \frac{{q_e}^2 k_2 t}{1 + q_e k_2 t} \text{(S4)}$$

where k_2 is the pseudo-second-order rate constant (mg g^{-1} min^{-1}), q_e the adsorption capacity at equilibrium (mg·g^{-1}) and q_t represents the amount adsorbed at time t (mg·g^{-1}) [3].

Text S4. Determination of thermodynamic parameters

The changes in standard Gibbs free energy (ΔG°), enthalpy (ΔH°), and entropy (ΔS°) were calculated using the following equations [5]:

$$ln\, k_c = \frac{\Delta S°}{R} - \frac{\Delta H°}{RT} \text{(S5)}$$

$$\Delta G° = \Delta H° - T\Delta S° \text{ (S6)}$$

where kc is the equilibrium constant, q_e the equilibrium adsorption capacity, R the gas constant (8.314 J·mol^{-1}·K^{-1}), and T the absolute temperature (K). The equilibrium constant was obtained using the following expression [6]:

$$k_c = \frac{q_e}{C_e} * \frac{m}{v} \text{(S7)}$$

where q_e is the equilibrium metal adsorption capacity (mg g^{-1}), Ce is the amount of metal remaining in the solution (mg L^{-1}) at equilibrium, m is the mass of adsorbent (g), and V is the volume of adsorbate solution (L). By using this equation, Kc becomes dimensionless. The slope and intercept of the Van't Hoff plot (ln Kc vs. 1/T) are used to estimate ΔH° and ΔS°, respectively [7].

Text S5. Average percentage error (APE)

The average percentage error (APE) was calculated using the following equation:

$$APE\ (\%) = \frac{\sum_{i=1}^{N} \left| \left(\frac{q_{exp} - q_{cal}}{q_{exp}} \right) \right|}{N} * 100 \text{ (S8)}$$

where q_{exp} and q_{cal} represent the experimental and calculated equilibrium amounts of Hg^{2+} adsorbed, respectively, while Ndenotes the number of measurements. The APE quantifies the level of agreement between the experimental results and the adsorption values predicted by the model.

Text S6. Description of the Langmuir isotherm

The most common adsorption model is the Langmuir model expressed as follows [8]:

$$q_e = \frac{q_m K_L C_e}{1 + K_L C_e} \text{(S9)}$$

In this model, C_e (mg L^{-1}) denotes the equilibrium concentration of the adsorbate, and q_e (mg g^{-1}) represents the amount adsorbed per unit mass of adsorbent. The parameter q_m (mg g^{-1}) corresponds to the maximum adsorption capacity of Hg^{2+}, that is, the amount required to form a complete monolayer. The affinity constant K_L (L m g^{-1}) reflects the equilibrium of the adsorption process. Consequently, by plotting C_e/q_e against C_e, q_m can be obtained from the slope of the line, whereas K_L is determined from its intercept.

The Langmuir isotherm can be further characterized by a dimensionless equilibrium parameter, known as the separation factor, defined as follows [9]:

$$R_L = \frac{1}{1 + K_L C_o} \text{(S10)}$$

According to the value of the separation factor (R), the isotherm shape can be categorized as linear (R = 1), unfavorable (R > 1), favorable (R < 1), or irreversible (R = 0) [10].

Text S7. Description of the Freundlich isotherm

The Freundlich isotherm can be studied using the following equation [11]:

$$q_e = K_F C_e^{1/n} \quad \text{(S11)}$$

The constant K_f is an approximate indicator of adsorption capacity, while n is a function of the strength of adsorption in the adsorption process.

Text S8. Description of the SIPS isotherm

The Sips isotherm is a combined form of Langmuir and Freundlich models deduced for predicting the adsorption in heterogeneous systems and circumventing the limitation of the rising adsorbate concentration associated with the Freundlich isotherm [12]. The Langmuir–Freundlich isotherm is given as [13]:

$$q_e = \frac{q_{MLF}(K_{LF} C_e)^{M_{LF}}}{1 + (K_{LF} C_e)^{M_{LF}}} \quad \text{(S12)}$$

where q_{MLF} indicates the Langmuir–Freundlich maximum adsorption capacity (mg g^{-1}), the heterogeneous surface's equilibrium constant is K_{LF}, M_{LF} is heterogenous parameter between 0 and 1, and C_e is the equilibrium concentration.

The Sips isotherm equation is characterized by the dimensionless heterogeneity factor (M_{LF}), which serves to describe the degree of heterogeneity of the system when n lies within the range 0–1. Specifically, when M_{LF} = 1, the Sips model reduces to the Langmuir equation, indicating a homogeneous adsorption process. In contrast, when M_{LF} < 1, the model reflects a heterogeneous adsorption process [14].

Text S9. Relative growth index (RGI)

The relative growth index (RGI) was determined using the following equation [15]:

$$RGI = \frac{SRL}{CRL} \quad (S13)$$

where SRL is the sample rootlet length, and CRL is the control rootlet length.

Table S1. Elemental composition of adsorbents by energy-dispersive X-ray spectroscopy (EDS).

Element	FAU-type Y	FAU-type X
O	46.2	44.0
Na	8.70	6.70
Al	17.3	23.2
Si	27.8	26.1

Table S2. Interplanar spacing in FAU-type Y and FAU-type X.

FAU-type Y						FAU-type X					
Plane	2Θ (°)	d_{hkl} (Å)	Plane	2Θ (°)	d_{hkl} (Å)	Plane	2Θ (°)	d_{hkl} (Å)	Plane	2Θ (°)	d_{hkl} (Å)
1 1 1	6,15	14,4	9 5 5	41,47	1,78	1 1 1	6,07	14,56	11 1 1	39,79	2,27
2 2 0	10,03	8,82	8 6 6	42,29	1,76	2 2 0	9,94	8,90	8 8 0	40,64	2,22
3 1 1	11,78	7,51	9 7 3	42,78	1,72	3 1 1	11,66	7,59	11 3 1	41,14	2,19
3 3 1	15,49	5,72	8 8 4	43,58	1,70	3 3 1	15,34	5,78	11 3 3	42,43	2,13
4 2 2	17,37	5,11	9 7 5	45,30	1,68	5 1 1	18,31	4,85	12 0 0	43,23	2,09
3 3 3	18,48	4,80	8 8 6	46,67	1,67	4 4 0	19,97	4,45	12 4 2	46,27	1,96
4 4 0	20,14	4,41	10 8 2	47,28	1,65	5 3 1	20,88	4,25	10 8 2	46,89	1,94

5 3 1	21,02	4,23	10 6 6	47,80	1,62	6 2 0	22,41	3,97	11 5 5	47,34	1,92
6 2 0	22,55	3,94	9 7 7	48,91	1,60	5 3 3	23,21	3,83	11 3 7	48,49	1,88
5 3 3	23,39	3,80	12 6 2	49,63	1,58	4 4 4	24,52	3,63	13 3 3	49,64	1,84
6 2 2	23,68	3,76	9 9 5	50,05	1,57	6 4 2	26,54	3,36	13 5 1	50,77	1,80
4 4 4	24,74	3,60	8 8 8	50,76	1,56	7 3 3	29,10	3,07	14 2 0	51,48	1,78
5 5 1	25,51	3,49	11 7 5	51,19	1,55	8 2 2	30,21	2,96	11 9 3	52,99	1,73
6 4 2	26,75	3,33	10 8 6	51,88	1,53	7 5 1	30,82	2,90	14 4 2	53,60	1,71
5 5 3	27,45	3,25	9 9 7	53,40	2,18	8 4 0	31,86	2,81	14 6 2	56,30	1,63
7 3 3	29,31	3,05	10 10 4	54,09	2,14	9 1 1	32,52	2,75	13 7 5	57,22	1,61
6 6 0	30,42	2,94	11 7 7	54,49	2,11	6 6 4	33,46	2,68	15 5 1	58,20	1,59
5 5 5	31,06	2,88	12 8 4	55,16	2,08	9 3 1	34,03	2,63	16 0 0	58,86	1,57
8 4 0	32,11	2,79	11 9 5	55,57	2,00	8 4 4	35,00	2,56	15 5 3	59,24	1,56
7 5 3	32,73	2,74	10 10 6	56,74	1,95	10 2 0	36,47	2,46	16 2 2	59,89	1,54
6 6 4	33,73	2,66	9 9 9	57,66	1,92	10 2 2	37,19	2,42	11 11 5	60,25	1,54
9 3 1	34,31	2,61	12 10 2	58,30	1,90						
8 4 4	35,28	2,54	11 9 7	58,68	1,86						
8 6 2	36,77	2,44	16 0 0	59,33	1,84						
6 6 6	37,49	2,40	13 9 3	59,70	1,82						
9 5 3	38,90	2,32	10 10 8	60,35	1,80						
7 7 5	40,12	2,25									

Table S3. X-ray fluorescence analysis of real-water samples (mass %).

Component	Real water
**H_2O**	99.88%

**Al**	0.074%
**Fe**	0.017%
**Ni**	0.0096%
**Cu**	0.0050%
**Zn**	0.0025%
**Cd**	0.0017%
**Sn**	0.0030%

Table S4. Elemental composition of adsorbents by X-ray fluorescence analysis.

Element	FAU-type Y	FAU-type X
Si	75.6	70.0
Al	24.4	30.0

Table S5. Microbiological analysis in Caquetá River. Conditions: Temperature 37°C, adsorbed dose 0.75 g L^{-1}, stirring rate 220 rpm.

**River water**	***E. coli*** **(CFU/100 mL)**	**Total coliforms (CFU/100 mL)**
Without doping Hg^{2+}	33	800
Without doping Hg^{2+} and treated (FAU-type X)	0	8
With Hg^{2+}	0	0
With Hg^{2+} and treated (FAU-type X)	0	0

Table S6. Classification of RGI values [15].

Inhibition of elongation	0 < x < 0.8
No significant effects	0.8 < x < 1.2

Stimulation of root elongation	x > 1.2, where *x* represents the calculated RGI value

Table S7. Composition of saline solution.

Compound	Formula	Concentration (mg L^{-1})
Sodium bicarbonate	$NaHCO_3$	64.75
Calcium chloride dihydrate	$CaCl_2·2H_2O$	294.0
Magnesium sulfate heptahydrate	$MgSO_4·7H_2O$	123.25
Potassium chloride	KCl	5.75

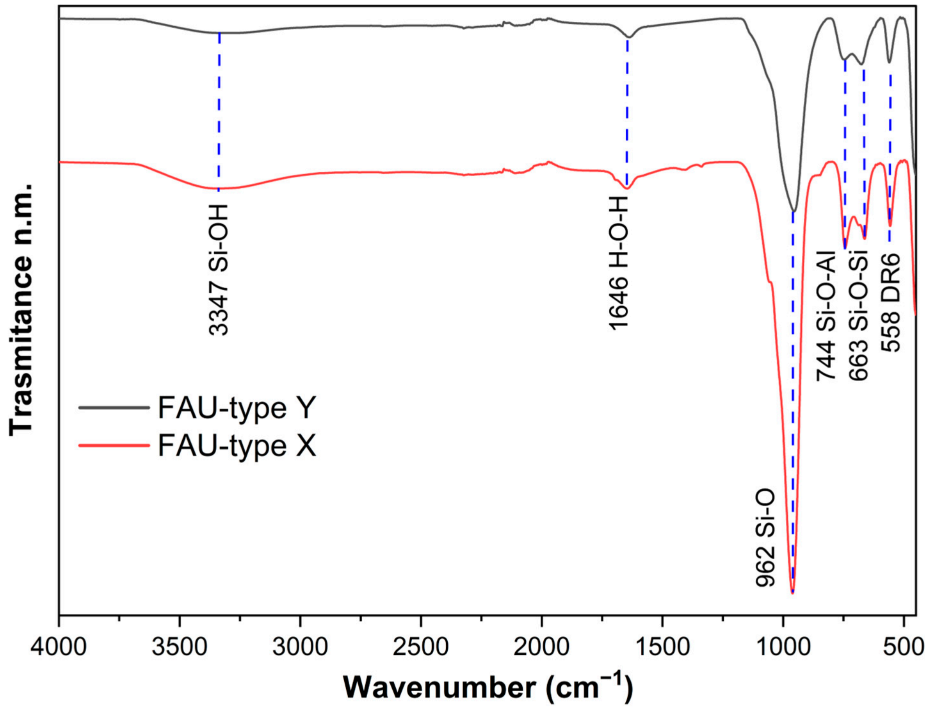

Figure S1. FTIR analysis of Zeolites FAU-type Y and FAU-type X.

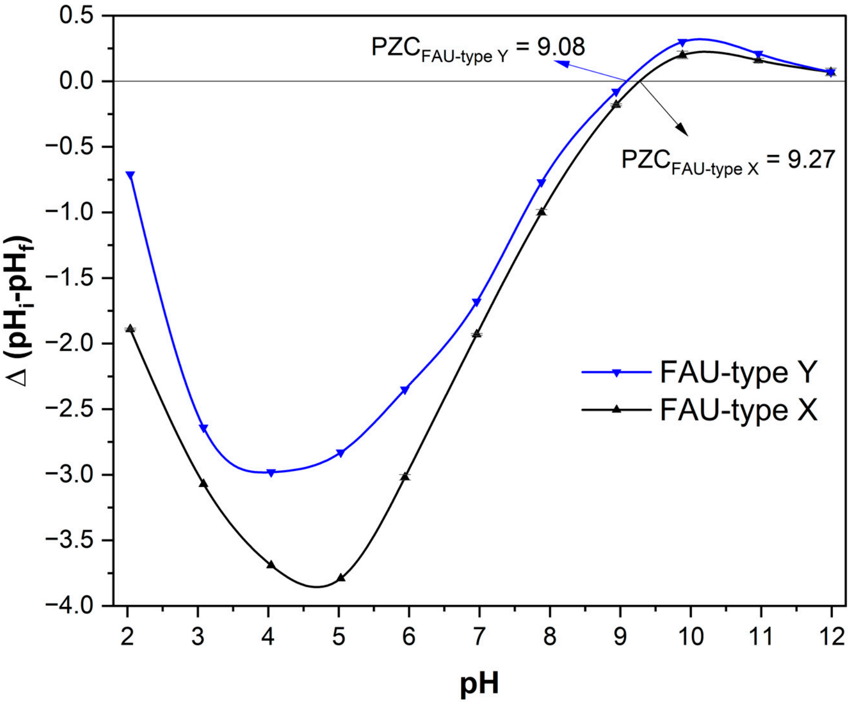

Figure S2. Point of charge zero. Conditions: NaCl 0.1 M, adsorbent dose 1.0 g L^{-1}, stirring rate 200 rpm, temperature 25 °C.

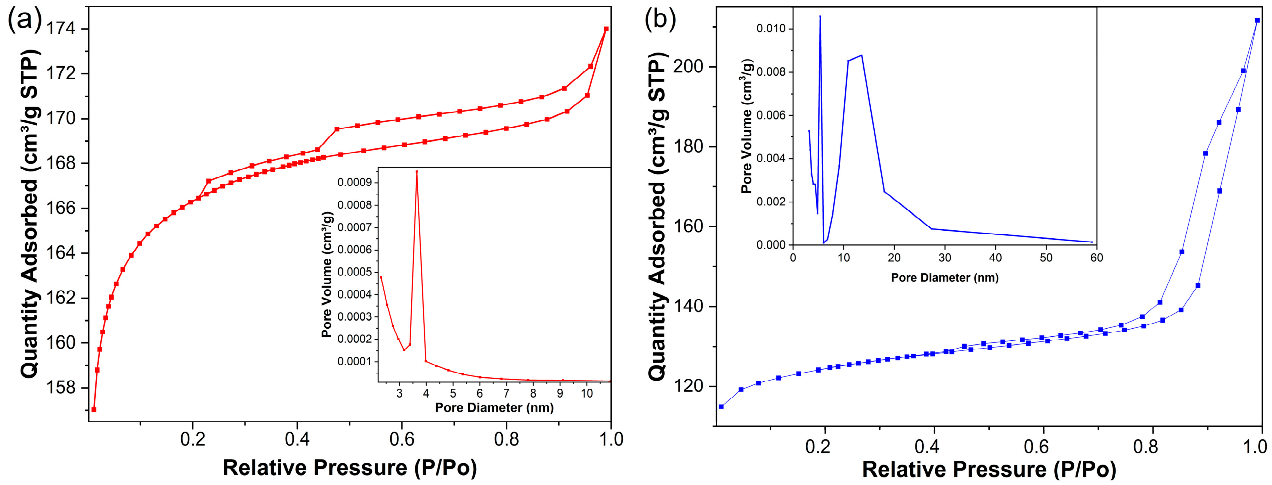

Figure S3. N_2 adsorption–desorption isotherms with inset pore size distribution for: (a) FAU-type Y and (b) FAU-type X.

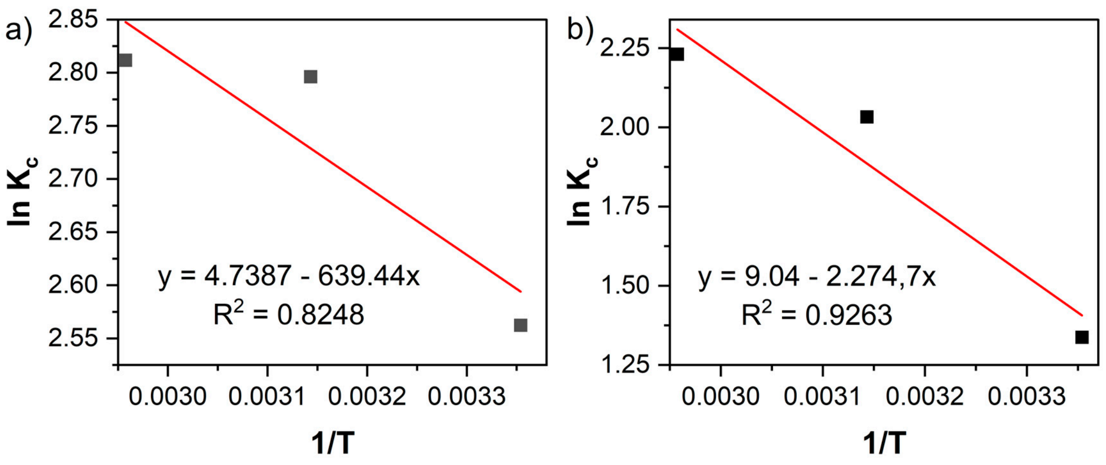

Figure S4. ln K_c vs. 1/T to obtain the thermodynamic parameters in the Hg^{2+} adsorption. Conditions: Hg^{2+} concentration 1.0 mg L^{-1}, dose 1.0 g L^{-1}, pH 6.8. time 12 h, temperature 25-65 °C.

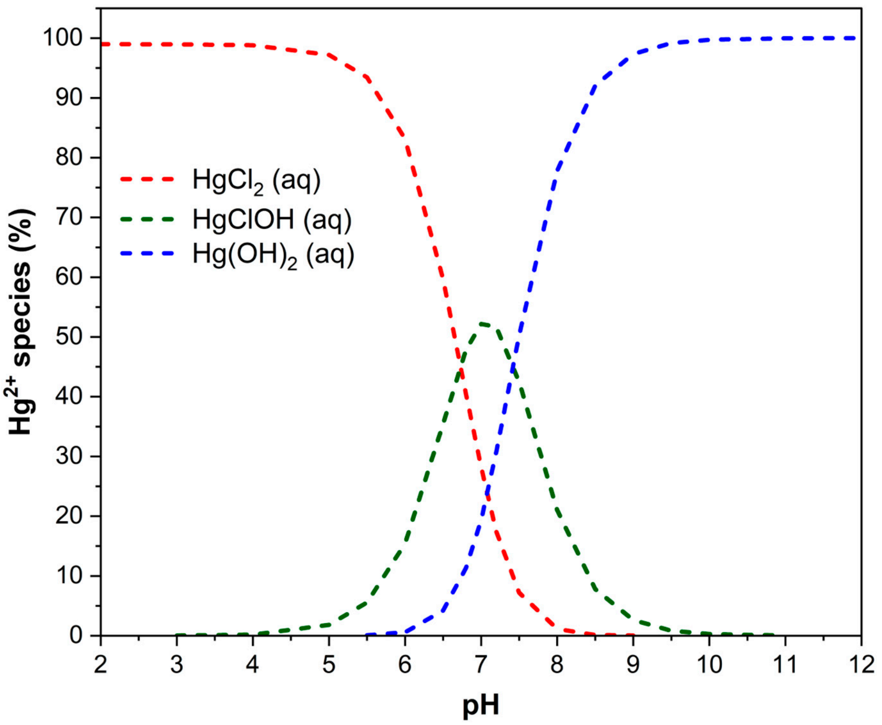

Figure S5. Distribution of Hg^{2+} species diagram using the Visual MINTEQ program. Conditions: [Hg] = 0.90 mg L^{-1}, [HCl] = 0.001 mol L^{-1}.

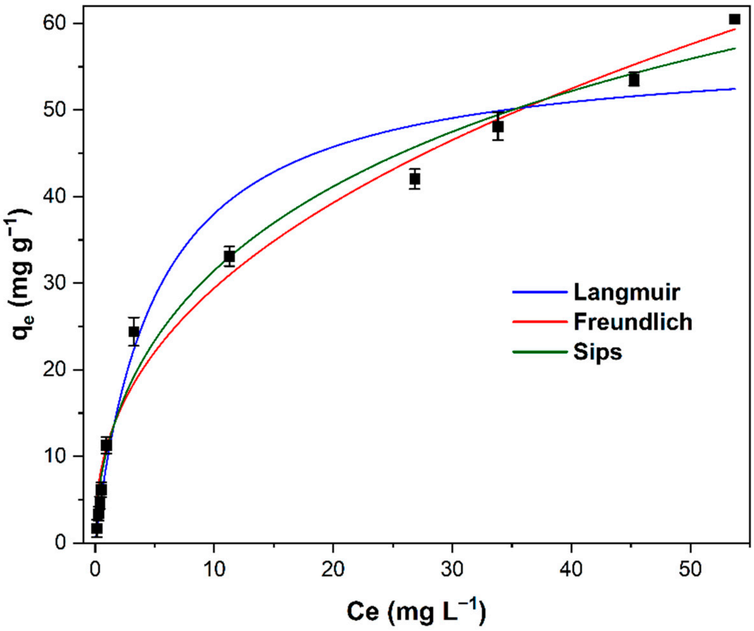

Figure S6. Langmuir, Freundlich and SIPS isotherms. Conditions: pH 6.8. stirring rate 270 rpm, time 24 h, adsorbent dose 0.75 g L^{-1} temperature 25 °C, initial concentration Hg^{2+} 1-100 mg L^{-1}.

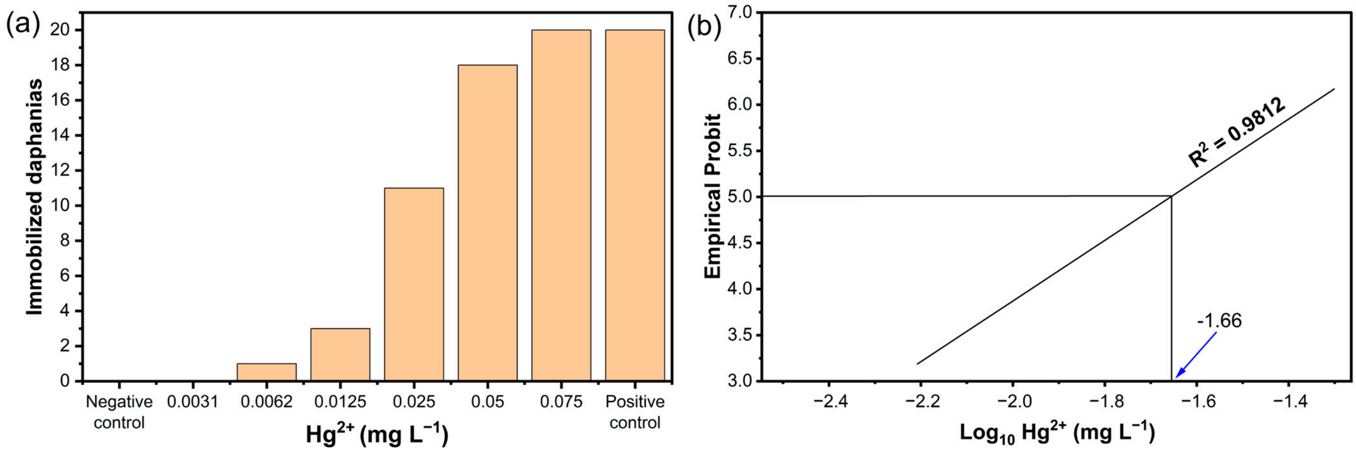

Figure S7. Ecotoxicological assessment: (a) Immobility of *Daphnia magna*. (b) Probit at 24 h. Conditions: pH 6.5, temperature 22 °C, distilled water with saline solution (Table S7).

References

1. Marchiori, R. Mathematical Fundamentals of Nanotechnology. In *Nanostructures*; de Oliveira Jr, O., Da Róz, A.L., Ferreira, M., de Lima Leite, F., Eds.; William Andrew Publishing, 2017; pp. 209–232.
2. Mercado-Borrayo, B.M.; Schouwenaars, R.; Litter, M.I.; Montoya-Bautista, C. V.; Ramírez-Zamora, R.M. Metallurgical Slag as an Efficient and Economical Adsorbent of Arsenic. In *Water Reclamation and Sustainability*; Ahuja, S., Ed.; Elsevier, 2014; pp. 95–114.

3. Zafar, S.; Khalid, N.; Daud, M.; Mirza, M.L. Kinetic Studies of the Adsorption of Thorium Ions onto Rice Husk from Aqueous Media: Linear and Nonlinear Approach. *The Nucleus* **2015**, *52*, 14–19.
4. Ebelegi, A.N.; Ayawei, N.; Wankasi, D.; Ebelegi, A.N.; Ayawei, N.; Wankasi, D. Interpretation of Adsorption Thermodynamics and Kinetics. *Open J. Phys. Chem.* **2020**, *10*, 166–182.
5. Lima, E.C.; Hosseini-Bandegharaei, A.; Moreno-Piraján, J.C.; Anastopoulos, I. A Critical Review of the Estimation of the Thermodynamic Parameters on Adsorption Equilibria. Wrong Use of Equilibrium Constant in the Van't Hoof Equation for Calculation of Thermodynamic Parameters of Adsorption. *J. Mol. Liq.* **2019**, *273*, 425–434.
6. Taguba, M.A.M.; Ong, D.C.; Ensano, B.M.B.; Kan, C.C.; Grisdanurak, N.; Yee, J.J.; de Luna, M.D.G. Nonlinear Isotherm and Kinetic Modeling of Cu(II) and Pb(II) Uptake from Water by MnFe2O4/Chitosan Nanoadsorbents. *Water.* **2021**, *13*, 1662.
7. Sadegh Mazloom, M.; Hemmati-Sarapardeh, A.; Husein, M.M.; Shokrollahzadeh Behbahani, H.; Zendehboudi, S. Application of Nanoparticles for Asphaltenes Adsorption and Oxidation: A Critical Review of Challenges and Recent Progress. *Fuel* **2020**, *279*, 117763.
8. Khandoozi, S.; Sharifi, A.; Riazi, M. Enhanced Oil Recovery Using Surfactants. In *Chemical Methods*; Hemmati-Sarapardeh, A., Schaffie, M., Ranjbar, M., Dong, M., Li, Z., Eds.; Gulf Professional Publishing, 2022; pp. 95–139.
9. Kanwal, S.; Devi, P.; Ahmed, Z.; Qambrani, N.A. Adsorption Isotherm, Kinetic and Thermodynamic Studies for Adsorption of Fluoride on Waste Marble Powder. *Desalination Water Treat.* **2024**, *319*, 100441.
10. Molina-Calderón, L.; Basualto-Flores, C.; Paredes-García, V.; Venegas-Yazigi, D. Advances of Magnetic Nanohydrometallurgy Using Superparamagnetic Nanomaterials as Rare Earth Ions Adsorbents: A Grand Opportunity for Sustainable Rare Earth Recovery. *Sep. Purif. Technol.* **2022**, *299*, 121708.
11. Tzabar, N.; ter Brake, H.J.M. Adsorption Isotherms and Sips Models of Nitrogen, Methane, Ethane, and Propane on Commercial Activated Carbons and Polyvinylidene Chloride. *Adsorption* **2016**, *22*, 901–914.
12. Belhachemi, M.; Addoun, F. Comparative Adsorption Isotherms and Modeling of Methylene Blue onto Activated Carbons. *Appl. Water Sci.* **2011**, *1*, 111–117.
13. Kumara, N.T.R.N.; Hamdan, N.; Petra, M.I.; Tennakoon, K.U.; Ekanayake, P. Equilibrium Isotherm Studies of Adsorption of Pigments Extracted from Kuduk-Kuduk (Melastoma Malabathricum L.) Pulp onto TiO2 Nanoparticles. *J. Chem.* **2014**, *2014*, 468975.
14. De Almeida, M.; Machado, M.R.; Costa, G.G.; de Oliveira, G.A.R.; Nunes, H.F.; Maciel Costa Veloso, D.F.; Ishizawa, T.A.; Pereira, J.; Ferreira de Oliveira, T. Influence of Different Concentrations of Plasticizer Diethyl Phthalate (DEP) on Toxicity of Lactuca Sativa Seeds, Artemia Salina and Zebrafish. *Heliyon* **2023**, *9*, e18855.
15. Silva, A.M. dos S.; Figueirêdo, L.P. de; Araújo, G.M.; Aragão, J.S. Phytotoxicity of Domestic Effluent Before and After Treatment by Stabilization Ponds. *Ecotoxicology and Environmental Contamination* **2024**, *19*, 50–60.
